# p38 MAPK regulates PKAα and CUB-serine protease in *Amphibalanus amphitrite* cyprids

**DOI:** 10.1038/srep14767

**Published:** 2015-10-05

**Authors:** Gen Zhang, Li-Sheng He, Yue Him Wong, Ying Xu, Yu Zhang, Pei-Yuan Qian

**Affiliations:** 1 Environmental Science Programs and Division of Life Science, School of Science, the Hong Kong University of Science and Technology, Clear Water Bay, Kowloon, Hong Kong SAR, P. R. China; 2 Sanya Institute of Deep-sea Science and Engineering, Chinese Academy of Science, No. 62, Fenghuang Road, Sanya, Hainan, P. R. China, 572000; 3 Shenzhen Key Laboratory of Marine Bioresource and Eco-environmental Science, College of Life Science, Shenzhen University, Shenzhen, P. R. China, 518060

## Abstract

The MKK3-p38 MAPK pathway has been reported to mediate larval settlement in *Amphibalanus* (=*Balanus*) *amphitrite*. To clarify the underlying molecular mechanism, we applied label-free proteomics to analyze changes in the proteome of cyprids treated with a p38 MAPK inhibitor. The results showed that the expression levels of 80 proteins were significantly modified (*p* < 0.05). These differentially expressed proteins were assigned to 15 functional groups according to the KOG database and 9 pathways were significantly enriched. Further analysis revealed that p38 MAPK might regulate the energy supply and metamorphosis. Two potential regulatory proteins, CUB-serine protease and PKAα, were both down-regulated in expression. CUB-serine protease localized to postaxial seta 2 and 3, as well as the 4 subterminal sensilla in the antennule. Importantly, it was co-localized with the neuron transmitter serotonin in the sections, suggesting that the CUB-serine protease was present in the neural system. PKAα was highly expressed during the cyprid and juvenile stages, and it was co-localized with phospho-p38 MAPK (pp38 MAPK) to the cement gland, suggesting that PKAα might have some functions in cement glands. Overall, p38 MAPK might regulate multiple functions in *A. amphitrite* cyprids, including the energy supply, metamorphosis, neural system and cement glands.

The barnacle *Amphibalanus amphitrite* is a cosmopolitan fouling organism and is widely used as a model organism in biofouling and intertidal ecology research. The molecular mechanism underlying larval attachment and metamorphosis has always been a hot topic of antifouling and ecological research. In the past decade, some pathways, proteins and molecules have been suggested to participate in the larval settlement of this species. A glycoprotein, namely settlement-inducing protein complex (SIPC), was isolated from adult barnacles and induces larval settlement^1^. Signaling molecules, including cAMP^2^, NO^3^ and cGMP^4^, regulate larval settlement in *A. amphitrite*. Some proteins related to energy and metabolism, such as acetyl-CoA acetyltransferase, fructose-bisphosphate aldolase, fructose 1,6-bisphosphatase, glucose-6-phosphate isomerase, glucosamine-6-phosphate isomerase 1 and mannose-6-phosphate isomerase, may regulate fat digestion and then the energy supply during larval settlement^5^. Calmodulin, myosin light chain kinase^6^ and cement proteins^6^,^7^ have also been reported to be involved in larval settlement. Although these independent studies identified various key components that regulate larval settlement, a global view of the underlying molecular mechanism is still lacking.

Mitogen-activated protein kinase (MAPK) pathways, including p38 MAPK, JNK and ERK, are conserved among different animals. Among them, p38 MAPK is directly activated by MAPK kinase 3/6 (MAPKK3/6 or MKK3/6) through dual phosphorylation of the activation sites, and activation of these MKKs is mediated by MAPKK kinase (MAPKKK or MKKK) also through dual phosphorylation^8^. To date, approximately 200–300 molecules have been reported to be substrates of p38 MAPK, including transcription factors and regulators of chromatin remodeling, protein degradation and localization, mRNA stability, endocytosis, apoptosis, cytoskeletal dynamics, cell migration, respiratory burst activity, chemotaxis, granular exocytosis and adherence (summarized in^8^,^9^), and they regulate most signal transduction pathways.

In *A. amphitrite*, p38 MAPK participates in larval settlement^10^, and its involvement is specifically mediated by MKK3^11^. Inhibition of p38 MAPK using SB203580 was found to effectively block larval settlement but did not affect their ability to explore the substratum^10^. Both MKK3 and p38 MAPK were localized in the antennules of *A. amphitrite* cyprids, which are responsible for surface exploration and cement secretion. Moreover, the MKK3-p38 MAPK pathway mediates the stimulatory effects of SIPC on larval settlement^10^,^11^, which suggests that the MKK3-p38 MAPK pathway might play a pivotal role in signal detection and/or transduction during larval exploration of the substratum. However, the molecular mechanism by which the p38 MAPK pathway participates in larval settlement remains unknown.

Proteomics methods are powerful tools to narrow down candidates in screens for target proteins. These methods have been successfully applied to study the effects of poly-ether B^12^, butenolide^13^ and meleagrin^14^ on changes in the proteome of *A. amphitrite* cyprids. In the present study, to further clarify the roles of p38 MAPK in larval settlement, we investigated changes in the proteome of *A. amphitrite* cyprids in response to treatment with the p38 MAPK specific inhibitor (SB203580) using label-free quantitative proteomics. Then, we further characterized the target proteins, through motif analysis and protein localization, to determine the potential functions of the candidate proteins.

## Results

### Proteome changes in response to SB203580 treatment

**Figure 1:**
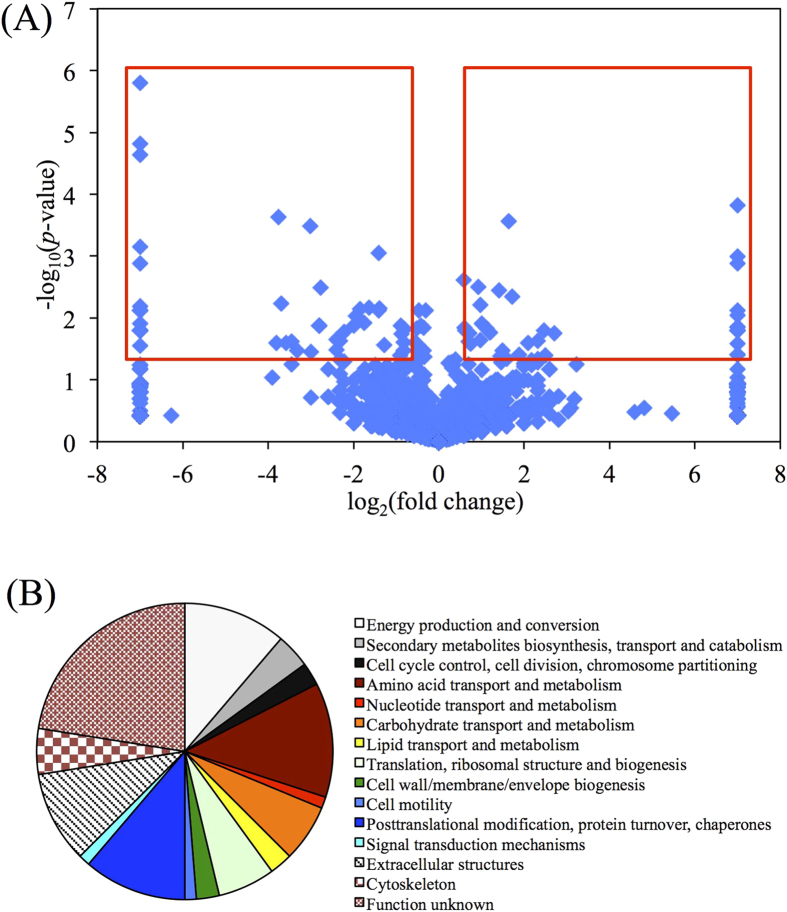
An overview of significantly differentially expressed proteins. (**A**) A volcano plot showing the distribution of fold change and *p*-value of all proteins. The vertical axis indicates –log_10_(*p*-value) and the horizontal axis indicates log_2_(fold change). The red rectangles include proteins with the fold change higher than 1.2 or lower than 0.83 with *p* < 0.05. Dots out of the range of ±7 at the horizontal axis were plotted at the line of ±7. (**B**) Functional grouping of proteins that were significantly changed in response to SB203580 treatment.

Trans-Proteomics Pipeline (TPP)^15^, a software suite for the analysis of MS/MS-based proteomics data, was used to compare the spectral counts of each protein between SB203580 (20 μM) treated cyprids and the control. TPP analysis identified 1502 ± 121 (mean ± SE) and 1450 ± 98 proteins in the treatment and the control, respectively. No significant differences in the number of proteins were detected between the SB203580 treatment and the control (Student’s t-test; *p* > 0.05). Student’s t-tests were performed on the spectral counts of each protein between the SB203580 treatment group and the control. Proteins with statistical difference (*p* < 0.05) and a fold change of >1.2 or <0.83 (which corresponded to 95% of confidence level based on the comparison of three biological replicates), were considered to be significantly differentially expressed^14^ (Fig. 1A). Finally, 80 proteins were significantly changed in the SB203580 treatment group compared with the control (Student’s t-test; *p* < 0.05). Among them, 34 were significantly up-regulated in expression and 46 were significantly down-regulated (*p* < 0.05; Supplementary Table. S1).

Significantly changed proteins were categorized into different functional groups by a BLAST analysis against the KOG database^16^. The results showed that 62 of the 80 proteins were categorized into 14 known functional groups and the remaining 18 proteins were included in the functional group designated as unknown (Fig. 1B). Among the 62 proteins, approximately 53% were assigned to the following four groups: 1). energy production and conversion; 2). amino acid transport and metabolism; 3). post-translational modification, protein turnover and chaperones, and 4). extracellular structures (Fig. 1B). The first 2 groups (energy production and conversion, amino acid transport and metabolism) were the most highly expressed, as they had the highest number of total spectral counts, despite the function unknown group (Table. 1).

The enriched pathways were analyzed using the KOBAS 2.0^17^. The differentially expressed proteins were mainly involved in 9 pathways. The first one, namely metabolism pathways, included 25 proteins. Each of the other 8 pathways included 3–6 proteins (Table. 2).

The proteomic results showed that both CUB (complement subcomponents Clr/Cls, Uegf, Bmp1)-serine protease (CUB-SP) and cAMP-dependent protein kinase type I-α regulatory subunit (PKAα) were decreased in expression in response to SB203580 treatment (Supplementary Table. S1). CUB-SP is potentially involved in signal detection and transduction^18^ and cAMP has been reported to regulate the larval settlement of barnacle^2^. These two proteins might play regulatory roles during the mediation of p38 MAPK on larval settlement. To validate the proteomic data and further investigate the underlying mechanism of p38 MAPK, antibodies were raised using rabbits to characterize these two proteins.

### Characterization of CUB-serine protease

**Figure 2:**
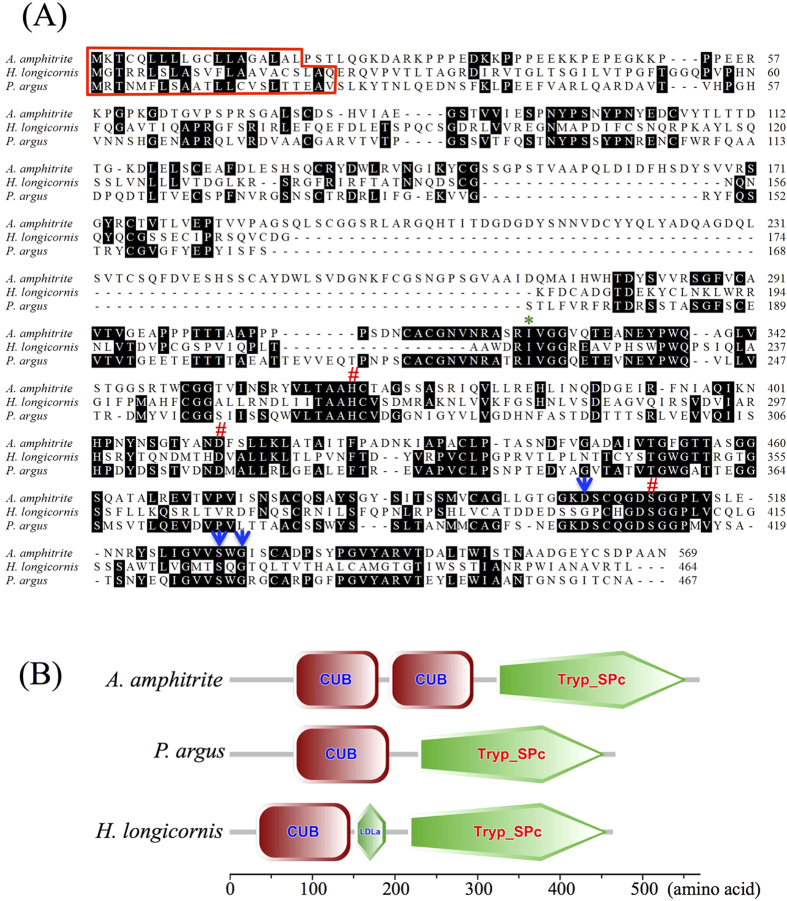
Characterization of the amino acid sequence of CUB-serine protease. (**A**) Amino acid sequence alignment of the CUB-serine protease from *Amphibalanus amphitrite*, *Haemaphysalis longicornis* (BAD21298.1) and *Panulirus argus* (AF357226.1). The cleavage site (Ile) is labeled with a green asterisk. The three active sites (His, Asp and Ser) and three substrate-binding sites (Asp, Ser and Gly) in the serine protease domain are marked with red pound signs and blue arrows, respectively. The predicted signal peptides are boxed with a red line. (**B**) Motif analysis of CUB-serine protease. CUB: complement C1r/C1s, Uegf, Bmp1 domain; Tryp_SPc: Trypsin-like serine protease domain; LDLa: low-density lipoprotein receptor domain class A.

The CUB-serine protease from *A. amphitrite* (Aa-CUB-SP) was found to be similar to CUB-SP from the hard tick *Haemaphysalis longicornis* (Hl-CUB-SP) and the lobster *Panulirus argus* (Pa-CUB-SP), with an identity of 23.47% and 40.37%, respectively (Fig. 2A). The Aa-CUB-SP protein consisted of 569 amino acid residues. It was 205 and 202 residues longer than Hl-CUB-SP and Pa-CUB-SP, respectively. Motif scanning using SMART website tools (http://smart.embl-heidelberg.de) revealed that Pa-CUB-SP and Hl-CUB-SP included only one CUB domain and one trypsin-like serine protease domain, but Aa-CUB-SP contained two CUB domains and one serine protease domain (Fig. 2B). The identity between these two CUB domains was 47.17%, with the similarity of 61.32% (Fig. S1).

Similarly to Pa-CUB-SP and Hl-CUB-SP, the signal peptide prediction using the SignalP 4.1 Server (http://www.cbs.dtu.dk/services/SignalP/) suggested that the segment consisting of the first 19 amino acid residues was a signal peptide in Aa-CUB-SP (Fig. 2A and Fig. S2). In the serine protease domain, the cleavage site (Ile), three active sites (His, Asp and Ser) and three substrate-binding sites (Asp, Ser and Gly) were all conserved in Aa-CUB-SP (Fig. 2A). If Aa-CUB-SP was cleaved just after Ile-324, the N-terminal portion, which contains two CUB domains (without a signal peptide), was estimated to be 32.62 kDa, while the C-terminal portion, which includes the SP domain, was approximately 25.31 kDa.

**Figure 3:**
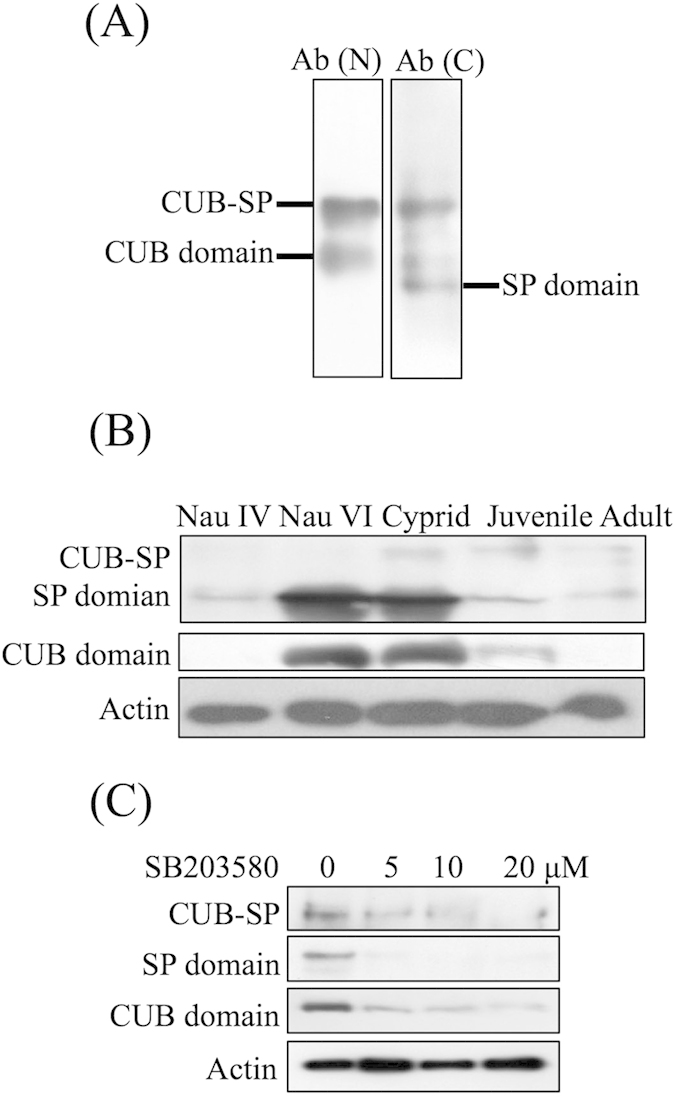
Characterization of CUB-Serine protease expression. (**A**) Two bands corresponding to 58 and 33 kDa were detected using an antibody against the CUB domain. Two bands corresponding to 58 and 25 kDa were detected using an antibody against the serine protease domain. Ab(N): antibody raised against the N-terminus of the CUB-serine protease (CUB domain). Ab(C): antibody raised against the C-terminus of the CUB-serine protease (serine protease domain). (**B**) Western blot showing that the CUB-serine protease was highly expressed in nauplius VI and the cyprid stages. Three bands, including the intact protein, CUB domain and serine protease domain of the CUB-serine protease, were visualized. The signals of the isolated CUB and serine protease domains were much stronger than that of the intact protein. Nau: nauplius. (**C**) Western blot showing that 5, 10 and 20 μM of SB203580 decreased the level of the intact protein as well as the isolated CUB and serine protease domains of the CUB-serine protease.

The antibody raised against the *A. amphitrite* CUB domain detected two bands in the Western blot analysis corresponding to 58 and 33 kDa. The antibody against the SP domain also detected two bands that were approximately 58 and 25 kDa (Fig. 3A), suggesting that the CUB and SP domains might be expressed as a single protein and then separated by cleavage at Ile-324 in Aa-CUB-SP.

Among the developmental stages of *A. amphitrite*, the results of the Western blot analysis showed that both isolated CUB and SP domains were highly present in the nauplius VI and cyprid stages (Fig. 3B). The intact CUB-SP protein displayed a higher expression level in the cyprid, juvenile and adult stages, compared to the nauplius IV and VI stages (Fig. 3B).

The Western blot results showed that 5, 10 and 20 μM SB203580 treatments decreased the levels of intact protein and of isolated CUB and SP domains of CUB-SP (Fig. 3C).

**Figure 4:**
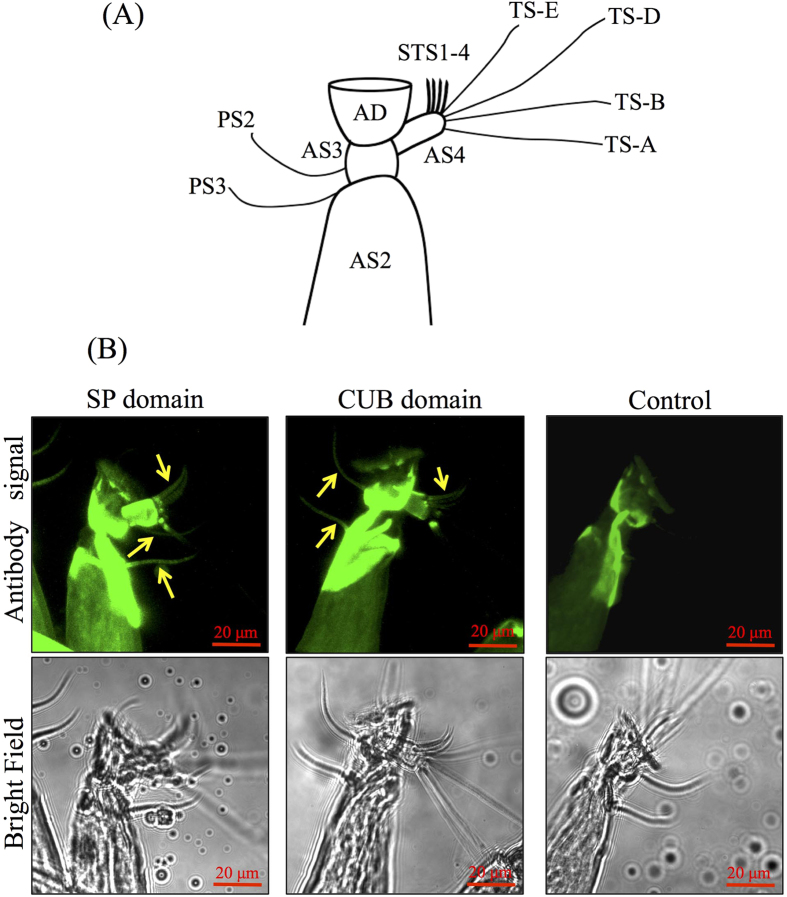
Immunostaining of whole mount cyprids revealed that the CUB-serine protease was localized to postaxial seta 2 and 3 as well as the four subterminal sensilla. (**A**) Schematic representation of a cyprid antennule. AD: attachment disc; AS2-4: antennulary segments 2–4; STS1-4: subterminal sensilla 1–4; TS-A, B, D and E: terminal sensilla A, B, D and E; PS2 and 3: postaxial seta 2 and 3. (**B**) Localization of the CUB-serine protein using antibodies against the CUB and the serine protease domains. The fluorescent signals from a set of Z-stack images were combined. The results showed that the CUB-serine protease localized to PS2, PS3 and four STS, which are indicated by yellow arrows.

**Figure 5:**
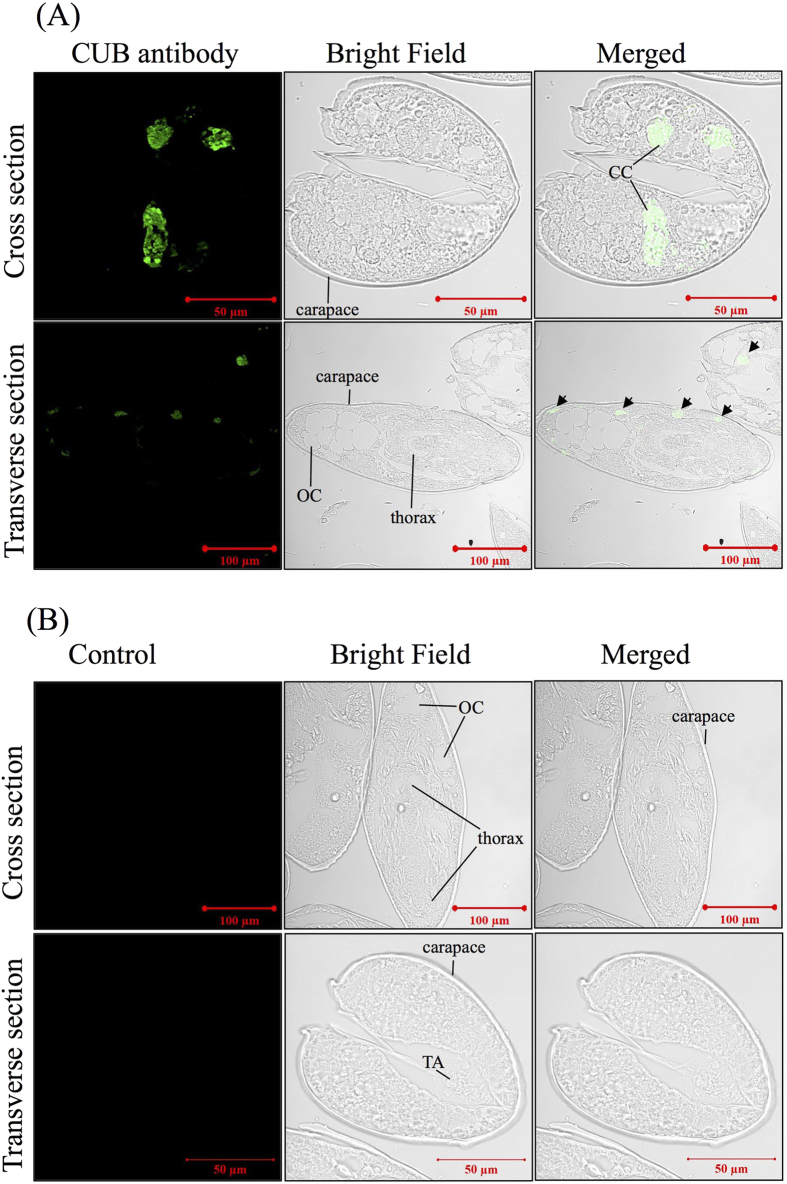
Immunostaining of the CUB-serine protease in cyprid sections. For each target protein, a transverse section and a longitudinal section are shown. (**A**) Immunostaining of CUB-serine protease showed signals at circumesophageal connectives in the cross sections, and at the nerve fibers nearby body wall in the transverse sections (Black arrows). (**B**) Control. Samples were incubated directly with the secondary antibody without any primary antibodies. No obvious singles were observed. TA: thoracic appendage; CC: circumesophageal connective; CG: cement gland; OC: oil cell; CE: compound eye.

Separate applications of the antibodies raised against the CUB and SP domains revealed consistent immunostaining results. Compared with the control, the signal for CUB-SP was visualized in the two postaxial setae (2 and 3) and four subterminal sensilla (1–4) (Fig. 4). In the sectioned cyprids, similar distributions of the signal were detected for both CUB-SP and serotonin (5-hydroxytryptamine, 5-HT). In the cross sections, strong signals were found symmetrically in the central region of the body (Fig. 5). These were neither sections across the brain nor sections across the posterior ganglion. Based on the morphological description of the nervous system and serotonin distribution in *A. amphitrite* cyprids^19^,^20^,^21^, the regions of signals were circumesophageal connectives that connected the brain and posterior ganglion. In the longitudinal sections, signal spots were symmetrically localized near the carapace (Fig. 5). The similar distribution pattern of CUB-SP and serotonin suggested that these regions of positive signal might be nerve fibers.

### Characterization of PKAα

PKAα from *A. amphitrite* showed a high similarity to those from other organisms (Supplementary Fig. S3).

**Figure 6:**
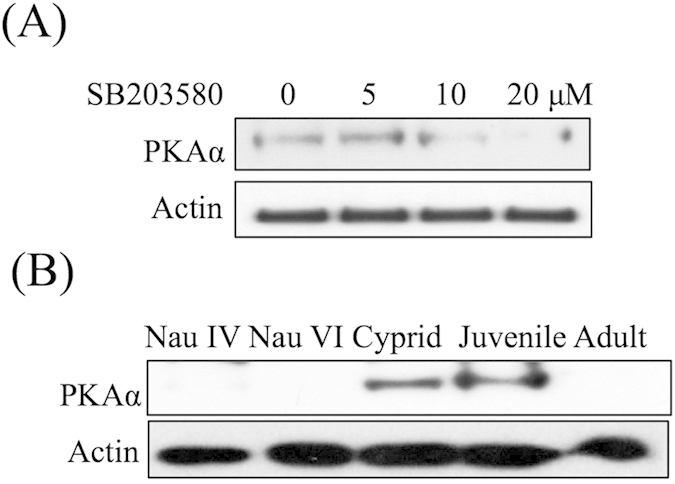
Characterization of PKAα expression. (**A**) Western blot showing that the level of PKAα decreased in response to 10 and 20 μM SB203580. (**B**) PKAα was highly expressed during the cyprid and juvenile stages.

Western blot analysis confirmed that the inhibition of p38 MAPK using SB203580 decreased the level of PKAα (Fig. 6A). Among the developmental stages, PKAα was highly expressed in the cyprid and juvenile stages (Fig. 6B).

**Figure 7:**
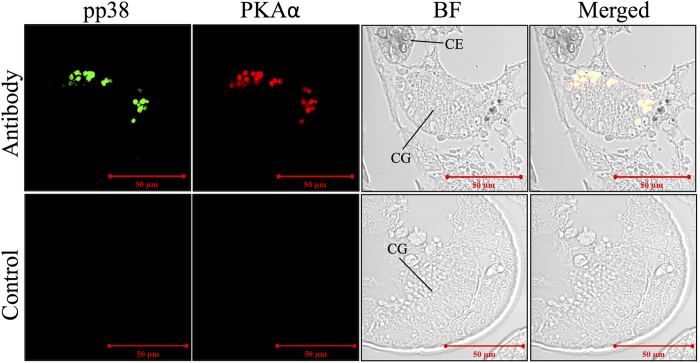
Immunostaining results against PKAα and pp38. Both PKAα and pp38 were localized to the collective duct of cement gland. CG: cement gland; CE: compound eye.

Co-immunostaining of phospho-p38 MAPK (pp38 MAPK) and PKAα revealed that both proteins were localized to the apical surface of cement glands (Fig. 7). Relatively weaker signals for PKAα were detected in the cells of the body wall that were immediately adjacent to the carapace (Supplementary Fig. S4).

## Discussion

SB203580 is a specific p38 MAPK inhibitor and widely used in a variety of cells or organisms. However, the evolutionary leap is rather great, and there are no guaranties that the specificity of SB203580 in mammals will be the same in barnacles. In particular, SB203580 might have other targets in barnacles, which have not been found in mammals. However, T103 in the ATP docking site that interacts with SB203580 was also conserved in the protein sequence of barnacle p38 MAPK^10^. In addition, the phosphorylation level of p38 MAPK was increased in response to SB203580 treatment due to the reverse feedback^11^, suggesting that SB203580 should have the activity of inhibiting p38 MAPK in barnacle.

Label-free quantitative proteomics is a reliable, versatile, and cost-effective alternative to labeled quantitation^22^. Compared with stable isotope labeling methods, label-free methods provide results that are closer to the expected values^22^. The present study, using the spectral counting method, detected 1502 ± 121 and 1450 ± 98 proteins in the treatment and the control, respectively, showing a higher protein coverage than those in previous studies of *A. amphitrite* cyprids that used 2D gel-based proteomics^13^,^23^ or iTRAQ labeling proteomics^14^.

### p38 MAPK Mediated Energy Metabolism

At cyprid stage, energy metabolism process (from digesting the lipids stored in oil cells to ATP synthesis and consumption) is crucial to larval settlement^24^,^25^. In the present study, SB203580 treatment inhibited ATP synthesis and consumption, as the level of mitochondrial ATP synthase (0.13 fold-change), vacuolar ATP synthase (0.70 fold-change) and V-type proton ATPase (0.78 fold-change) were all significantly decreased (*p* < 0.05), thus limiting the energy input for general metabolic processes and subsequent settlement in cyprids. Propionyl-CoA carboxylase^26^ and methylmalonyl CoA epimerase^27^ are important for fatty acid digestion. The up-regulation of these two enzymes (1.63 and 5.6 fold-change, respectively) suggested that blocking of p38 MAPK, which limited ATP synthesis, might accelerate the digestion of lipids by a reverse feedback mechanism. These findings were similar to the results from the cyprids treated with meleagrin, a potential antifouling compound, in which the process of fat digestion was also accelerated^14^. Therefore, it is possible that p38 MAPK might regulate the intermediate process between fatty acid digestion and subsequent ATP synthesis.

In addition, glycogen synthase and dihydrolipoyl dehydrogenase, which play roles in gluconeogenesis and lipogenesis, respectively, were both up-regulated in this study (2.66 and 5.15 fold-change, respectively). PKA has inhibitory effects on lipogenesis^28^. The down-regulation of PKAα in response to SB203580 treatment suggested that up-regulation of gluconeogenesis and lipogenesis might be meditated by the PKA pathway.

### p38 MAPK Regulated Larval Metamorphosis

During the transition from the cyprid to the juvenile, drastic morphological changes involve tissue degeneration and regeneration, such as the degradation of larval tissues and the formation of a shell structure^29^. In the present study, β-N-acetylglucosaminidase was down-regulated in expression (0.12 fold-change), blocking the breakdown of chitin^30^, and the expression of chorion peroxidase was also suppressed (spectral count was 0 in the treatment group), indicating that the hardening process of the exoskeleton might be blocked^31^. Furthermore, dumpy is a gigantic extracellular protein that is required to maintain tension at epidermal-cuticle attachment sites^32^. The decreased level of dumpy (extracellular protein) and cuticular proteins following SB203580 treatment also supported that p38 MAPK regulated larval metamorphosis.

Moreover, advillin-like protein may have a unique function in the morphogenesis of neuronal cells for the formation of ganglia^33^. The SB203580 treatment decreased the level of advillin-like protein, suggesting that the development of the neuron system might be affected. Down-regulated expression levels of two nuclear lamina formation-related proteins (GL10641 and lamin-C; 0.54 and 0.53 fold-change, respectively) and cytoskeleton-related proteins (myosin heavy chain, plectin-1 and CBN-CYK-1 protein; 0.38, 0.25 and 0.21 fold-change, respectively) suggested that the process of mitosis^34^ might be inhibited. Taken together, p38 MAPK was suggested to regulate larval metamorphosis.

### p38 MAPK Down-regulated the Expression of CUB-serine Protease

Previous studies have revealed diverse functions of CUB-SP in invertebrates, including food digestion^35^, the olfactory system^18^ and immunity^36^. CUB-SP is unlikely to participate in food digestion in *A. amphitrite*, as it was highly expressed at cyprid stage that is a non-feeding stage. Immunostaining results for CUB-SP revealed signals in the postaxial seta 2 and 3 as well as the subterminal sensilla 1-4. Postaxial setae and subterminal sensilla are claimed to function in detection of environmental signals during larval exploration^37^. It is interesting to further investigate whether CUB-SP participates in this process. Serotonin is a neurotransmitter and widely distributed in animals and plants^38^. Anti-serotonin antibodies have been successfully employed to visualize neural systems^39^,^40^. In the present study, colocalization of CUB-SP and serotonin further suggested that CUB-SP was present in the neural system.

### p38 MAPK Suppressed PKAα

The barnacle larval settlement rate correlates positively with the intracellular cAMP level^2^. PKA is activated by cAMP. Down-regulation of PKA should, to some extent, behave similarly to the decreased level of cAMP and inhibit larval settlement. SB203580 treatment inhibited larval settlement and depressed the expression level of PKAα, suggesting that the expression of PKAα might be regulated by p38 MAPK (directly or indirectly). Furthermore, the colocalization of PKAα and pp38 MAPK to cement glands further supported a mediation of the PKAα level by the p38 MAPK pathway. A weaker PKAα signal at the body wall suggested that the PKA pathway might participate in other processes, such as growth or molting in cyprids^5^.

## Conclusions

Using label-free proteomics, we analyzed changes in the proteome of *A. amphitrite* cyprids treated with 20 μM SB203580. The results suggested that p38 MAPK might regulate the energy supply and inhibit metamorphosis in cyprids. Moreover, p38 MAPK might moderate the expression of two potential regulatory proteins, CUB-serine protease and PKAα, which were present in the neural system and cement gland, respectively. The results of this study advanced our understanding of the molecular mechanism underlying p38 MAPK during the larval settlement of *A. amphitrite*.

## Materials and Methods

### Ethics Statement

No specific permit is needed for barnacle studies in Hong Kong. The location at which the barnacles were collected does not belong to any national parks, protected areas or private lands. There were no protected species in the sampling area, and no local laws or regulations were overlooked.

The protocol used herein for antibody production in rabbits was approved by the Department of Health of the Government of the Hong Kong Special Administrative Region (Ref. no: (14–39) in DH/HA&P/8/2/2 Pt.6) and the Animal Ethics Committee at the Hong Kong University of Science and Technology (Ref. no: 2014042). The methods were carried out in accordance with the approved guideline.

### Barnacle Collection and Larval Culture

PVC plates (20 × 30 cm) were hung in the intertidal area at Tso Wo Hang Pier in Sai Kung, Hong Kong (22°23'31.30"N, 114°17'18.34"E) for 3 months to allow natural colonization of *A. amphitrite*. To obtain barnacle larvae, the PVC plates were transported to the laboratory. After air-drying for 48 hours, adult barnacles were immersed in 0.22-μm-filtered seawater (FSW) to release the larvae. A light source was placed nearby to attract the larvae. Within 3 hours, all of the released nauplius larvae were collected, identified under a microscope and transferred to a new tank. Nauplius larvae were cultured in FSW at 30 °C with a diet of *Chaetoceros gracilis* Schutt at 1 × 10^6^ cells/ml. The culture medium was changed, and the larvae were fed daily until they transformed into cyprids.

### SB203580 Treatment

SB203580 was purchased from Santa Cruz Biotechnology, Inc. (Texas, USA). It was dissolved in DMSO to prepare a 20 mM stock solution and stored in −20 °C.

Once the larvae started to transform into cyprids, the larvae were filtered through a series of meshes (355, 280, 128, 110 and 61-μm pore-sized) to separate cyprids and nauplii every 3 hours. Cyprids collected from the same batch (<3 hours old) were used for further study. Cyprids were treated with 20 μM SB203580 for 24 hours and then harvested using 110-μm pore-sized mesh. Concurrently, cyprids treated with 0.1% DMSO were prepared as a control. Three biological replicates were collected for both the treatment and the control.

### Protein Extraction and PAGE separation

Protein extraction buffer (40 mM HEPES, 8 M Urea, 40 mM DTT, commercial protease and phosphatase inhibitor cocktails, Roche, Indianapolis, IN, USA, pH 7.4) was prepared just before use. Total protein was extracted by sonication for 3 × 20 sec at an amplitude of 20% and a pulse of 1 sec: 1 sec (QSonica Q125 sonicator, USA). Crude extracts were centrifuged at 15,000 g for 10 min at 4 °C. Supernatants containing the extracted proteins were separated for further analysis.

The protein concentration was determined using a Bradford protein assay kit (Bio-Rad, USA). For each sample, 60 μg of total protein was loaded into a 4–20% gradient precast PAGE gel (OKGel, Shanghai, China) to remove non-protein components. To visualize the protein bands, the gel was stained with coomassie blue G250 using an eStain^®^ 2.0 Protein Staining System (Genscript, Nanjing, China). For further trypsin digestion, the gel lane of each sample was cut into four fragments based on the molecular weight (>75 kDa, 75–48 kDa, 48–17 kDa and <17 kDa).

### In-gel Digestion

The gel fragments were cut into small pieces individually. To remove the coomassie blue and other impurities, the gel particles were washed in 50 mM NH_4_HCO_3_/ACN (acetonitrile; V:V = 1:1) for 15 min, dehydrated in 100% ACN for 10 min and rehydrated in 50 mM NH_4_HCO_3_ for 5 min. This process was repeated 2 times. After the final dehydration in 100% ACN, the gel particles were air-dried. Next, they were reduced in 10 mM DDT/25 mM NH_4_HCO_3_ at 56 °C for 45 min and alkylated in 55 mM iodoacetamide/25 mM NH_4_HCO_3_ at room temperature for 30 min. After another wash in 50 mM NH_4_HCO_3_/ACN (V:V = 1:1) for 15 min and ACN for 10 min, the gel particles were digested in 25 mM NH_4_HCO_3_ containing 5 μg/L trypsin (Sequencing grade, Promega) at 37 °C for 16 hours.

The digestion solution was collected, and the peptides residing within the gel particles were further recovered by soaking them in 50 mM NH_4_HCO_3_/ACN (V:V = 1:1) for 15 min with gentle sonication (Branson 5200, USA), and then 100% ACN for 15 min. All of the collected solution was pooled together and dried in a Centrivap (Labconco, USA). The peptide samples were cleaned up using C18 Ziptip Pipette tips (Millipore, USA).

### Mass Spectrometry Analysis

The peptide samples were analyzed using a LTQ mass spectrometer, which was interfaced with a nanoelectrospray ion source coupled to a Thermo Accela LC (Thermo Fisher Scientific, Bremen, Germany). For each gel fragment, 1 μg of digested sample was injected into the system. The peptides were first enriched using a trap column (Zorbax X300 SB-C18, 5 × 0.3 mm, 5 μm particle size) and then separated using a C18 column (Thermo Bio-Basic-18, 150 × 0.1 mm, 300 Å pore size, 5 μm particle size). The separation was conducted according to the following steps: the column was equilibrated with 2% solvent B (0.1% formic acid in acetonitrile) in solvent A (0.1% formic acid in water) for 14 min; after injection, the column was flushed with 2% solvent B for 2 min, 2% to 10% solvent B for 2 min, 10% to 32% solvent B for 60 min, 30% to 60% solvent B for 6 min, 60% to 80% solvent B for 2 min and finally, the column was maintained with 80% solvent B for 2 min. The mobile phase was changed from 80% to 2% solvent B in 2 min. In each MS^1^ spectrum, 10 MS^2^ from the top 10 intense ions were scanned, with the dynamic exclusion of 60 s. Precursor ions with a single charge were ignored in MS^2^. The normalized collision energy was set at 30%, and one microscan was acquired for each spectrum. For each gel fragment, two injections were analyzed as technical repeats.

### Label-free Quantification by Spectral Counting

The raw data generated from the LC-MS/MS were converted into. *mgf* format using the MM file converter 3.9 and then searched against a customized protein database derived from the transcriptome database of *A. amphitrite*^41^ using the OMSSA program^42^. Common contaminant protein sequences such as porcine trypsin, human keratin, and bovine serum proteins were added to the database. Decoy sequences for each protein were also included in the database using the decoyfasta script^43^. In OMSSA, the Th precursor tolerance was set at 2.1 and the permitted missed cleavage was 2. Carbamidomethylation of cysteine was included as a fixed modification, while oxidation of methionine, N-terminal protein acetylation, and N-terminal peptide pyroglutamate formation were set as variable modifications.

The .*pep.xml* files generated by OMSSA were further analyzed using the Trans-Proteomics Pipeline (TPP)^15^. The protein identification false discovery rates (FDR) were calculated using MAYU (integrated in the TPP)^44^, and FDR was set at 0.01. Proteins with spectral counts higher than (or equal to) 5 in either the treatment group or the control or both were retained for further analysis.

### Statistics

Three biological replicates (from collection of cyprids to MS/MS analysis) for both treatment and the control were performed. For a given biological replicate, results from 8 injections (4 gel fragments ×2 technical repeats) were combined. The spectral counts for each protein were compared between the three biological replicates of treatment and the control using the *t* test. Proteins with statistical difference (*p* < 0.05) and a fold change of >1.2 or <0.83 (which corresponded to 95% of confidence level based on the comparison of three biological replicates), were considered to be significantly differentially expressed.

### Antibody Generation

For CUB-serine protease (CUB-SP), one segment from the CUB domain (amino acids 78–178) and one from the serine protease domain (amino acids 398–498) were selected for antibody generation. These two segments were fused into the pET-28a and pGEX-4T vectors, respectively, for over-expression. His_6_-tagged protein was injected 4 times intracutaneously into the backs of New Zealand white rabbits at 3-week intervals. The first two injections were mixed with Freund’s complete adjuvant (Sigma, USA), and the last two injections were mixed with Freund’s incomplete adjuvant (Sigma, USA). Sera were collected from the rabbits at 1 week after the final injection, and antibody was purified using affinity purification with GST-tagged protein as the ligands.

Rabbit anti-pp38 MAPK (phospho-p38MAPK, Thr180/Tyr182) and mouse anti-β-actin, as well as HRP-linked goat anti-rabbit and anti-mouse IgG secondary antibodies were purchased from Cell Signaling Technology (USA). Mouse anti-PKAα/β and rabbit anti-serotonin was purchased from Santa Cruz Biotechnology, Inc. (USA) and Sigma Aldrich (USA), respectively. Alexa Fluor 488-labeled donkey anti-rabbit IgG and Alexa Fluor 594-labeled donkey anti-mouse IgG secondary antibodies were purchased from Life Technologies (USA).

### Western Blot Analysis

Cyprids treated with 5, 10 and 20 μM SB203580 for 24 hours were prepared for further detection of the expression levels of PKAα and CUB-SP. To characterize the expression pattern of PKAα and CUB-SP during different developmental stages, fresh larvae from nauplius IV, nauplius VI and cyprid stages as well as juveniles and adults were collected for Western blot analysis.

In general, 80 μg of total protein per sample was separated in a 4–20% gradient PAGE gel (OKGel, China) and then transferred onto a PVDF membrane (0.22-μm pore size, Millipore, USA). The membrane was blocked in 3% BSA in TBST, incubated with primary antibody (1:1,000 dilution) at 4 °C overnight, washed with TBST for 3 × 15 min, incubated with the secondary antibody (1:10,000 dilution) and washed again with TBST for 3 × 15 min. Signals were visualized using WesternBright peroxide chemiluminescent detection reagent (advansta, USA) and recorded using medical blue sensitive X-ray films.

### Immunofluorescence Imaging

Cyprids were relaxed in 0.37 M MgCl_2_ for 15 min and then fixed in 4% paraformaldehyde in PBS (pH 7.4) at 4 °C overnight. Both whole mount animals and sections of the cyprids were immunostained. After washing with PBS for 3 × 15 min, the cyprids were dehydrated in a gradient ethanol series (30%, 50%, 70%, 80%, 95% and 100%), incubated in xylene and finally embedded in paraffin. The cyprids in paraffin were cut into 4-μm-thick slices and mounted on glass slides. To rehydrate the cyprid sections, the slides were incubated in xylene followed by a gradient ethanol series (100%, 95%, 80% 70%, 50% and 30%) and then PBS.

For immunostaining, the cyprid sections were heated in 10 mM citric acid at 95 °C for 15 min in advance. Then, both whole mount animals and sections of the cyprids were treated with 0.5% Triton X-100 in PBS for 30 min. After blocking in 3% BSA/PBS solution for 2 hours at room temperature, the samples were incubated with primary antibody (anti-PKA, anti-pp38, anti-5-HT and anti-CUB-SP, 1:300 dilution, in 3% BSA/PBS solution) at 4 °C overnight and then washed with PBS for 3 × 15 min. The cyprid sections were then further incubated with florescent signal-linked secondary antibody (1:1,000 dilution, in 5% BSA/PBS solution) at 4 °C for 12 hours and washed with PBS for 3 × 15 min. After mounting with coverslip using FluorSave Reagent (Calbiochem, Canada), the cyprids were observed and recorded using a laser scanning confocal microscope (Zeiss, LSM 710, ZEN 2009 software, USA). Samples that were incubated with secondary antibody alone were served as a control.

## Additional Information

**How to cite this article**: Zhang, G. *et al*. p38 MAPK regulates PKAα and CUB-serine protease in *Amphibalanus amphitrite* cyprids. *Sci. Rep*. **5**, 14767; doi: 10.1038/srep14767 (2015).

## Supplementary Material

Supplementary Information

## Figures and Tables

**Table 1:** Total spectral counts of proteins involved in different KOG functional groups.

No.	Functional group	Number of proteins	Total spectral counts		p value
			Control	SB203580	
1	Function unknown	18	562.0 ± 25.7	416.7 ± 16.3	0.002*
2	Energy production and conversion	9	240.0 ± 8.8	326.7 ± 19.3	0.004*
3	Amino acid transport and metabolism	10	218.7 ± 9.0	107.3 ± 15.8	0.001*
4	Carbohydrate transport and metabolism	5	120.0 ± 5.7	79.3 ± 9.6	0.006*
5	Extracellular structures	8	116.0 ± 12.3	128.0 ± 0.0	0.150
6	Secondary metabolites biosynthesis, transport and catabolism	3	114.7 ± 4.5	76.7 ± 5.4	0.001*
7	Posttranslational modification, protein turnover, chaperones	9	114.7 ± 12.8	156.0 ± 8.5	0.015*
8	Translation, ribosomal structure and biogenesis	5	108.7 ± 2.9	115.3 ± 5.4	0.126
9	Cytoskeleton	4	53.3 ± 2.9	26.7 ± 2.2	0.000*
10	Cell cycle control, cell division, chromosome partitioning	2	27.3 ± 2.9	14.7 ± 0.8	0.004*
11	Lipid transport and metabolism	2	18.0 ± 1.4	34.7 ± 2.9	0.002*
12	Cell motility	1	15.3 ± 1.6	4.0 ± 2.4	0.005*
13	Signal transduction mechanisms	1	14.7 ± 0.8	10.0 ± 1.4	0.012*
14	Cell wall/membrane/envelope biogenesis	2	8.0 ± 2.4	7.3 ± 1.6	0.398
15	Nucleotide transport and metabolism	1	6.7 ± 2.2	0.7 ± 0.8	0.017*

The spectral counts among three biological replicates are represented as the mean ± SE.

^*^indicates significant difference between SB203580 treatment and control (*p* < 0.05).

**Table 2:** Enriched pathways of significantly changed proteins in response to SB203580 treatment.

ID	Pathway name	Input number	Background number	*P* value	Corrected *P* value	Contig input
hsa01100	Metabolic pathways	25	1213	0.0000	0.0003	CL17009.Contig1, CL11184.Contig1, CL4572.Contig1, Unigene11462, Unigene11511, CL14828.Contig1, CL15192.Contig1, Unigene10435, CL6274.Contig1, CL7745.Contig1, CL1569.Contig1, CL11285.Contig1, CL992.Contig1, CL7151.Contig1, CL4041.Contig1, CL1.Contig245, CL791.Contig1, CL16496.Contig1, Unigene30671, CL8680.Contig1, CL593.Contig2, CL4543.Contig1, CL4124.Contig1, CL593.Contig1, CL6901.Contig1
hsa00280	Valine, leucine and isoleucine degradation	5	44	0.0000	0.0012	CL6901.Contig1, CL4572.Contig1, Unigene11462, CL11285.Contig1, CL17009.Contig1
hsa00190	Oxidative phosphorylation	6	133	0.0005	0.0098	CL14828.Contig1, CL593.Contig2, CL791.Contig1, CL1569.Contig1, CL593.Contig1, Unigene11511
hsa00480	Glutathione metabolism	4	49	0.0006	0.0105	CL2356.Contig1, CL1199.Contig2, CL16496.Contig1, CL4955.Contig1
hsa01200	Carbon metabolism	5	106	0.0013	0.0194	CL6901.Contig1, CL4572.Contig1, Unigene11462, CL11285.Contig1, CL17009.Contig1
hsa00640	Propanoate metabolism	3	28	0.0014	0.0204	CL4572.Contig1, CL11285.Contig1, CL17009.Contig1
hsa00830	Retinol metabolism	4	64	0.0015	0.0211	CL7745.Contig1, CL304.Contig5, CL304.Contig4, CL4124.Contig1
hsa00591	Linoleic acid metabolism	3	29	0.0016	0.0214	CL7151.Contig1, CL15192.Contig1, CL4124.Contig1
hsa04610	Complement and coagulation cascades	4	69	0.0020	0.0246	CL1088.Contig2, CL7.Contig9, CL15271.Contig1, CL6481.Contig1

The pathway enrichment was assessed using KEGG pathway analysis. The background number indicates the number of proteins included in the pathway in KEGG database. The input number means the number of proteins found in this study matching with the pathway.
