# Supplementary material for: p38 MAPK regulates PKAα and CUB-serine protease in Amphibalanus amphitrite cyprids
Source: Sci Rep. 2015 Oct 5;5:14767. doi: 10.1038/srep14767 (PMC4593178; doi:10.1038/srep14767)
Supplement: Supplementary Information [file srep14767-s1.pdf]

p38 MAPK regulates PKAα and CUB-serine protease in *Amphibalanus amphitrite* cyprids

ZHANG Gen[1], HE Li-sheng[2], WONG Yue Him[1], XU Ying[3], ZHANG Yu[3], QIAN Pei-yuan[1*]

Supplementary information

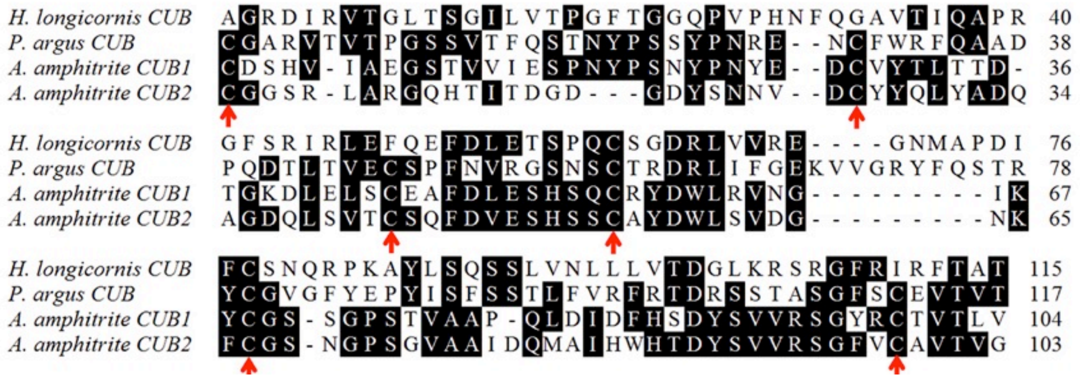

Figure S1. Amino acid sequence alignment of the CUB domain from *A. amphitrite*, *H. longicornis* and *P. argus*. Six conserved cysteine residues are labeled with red arrows.

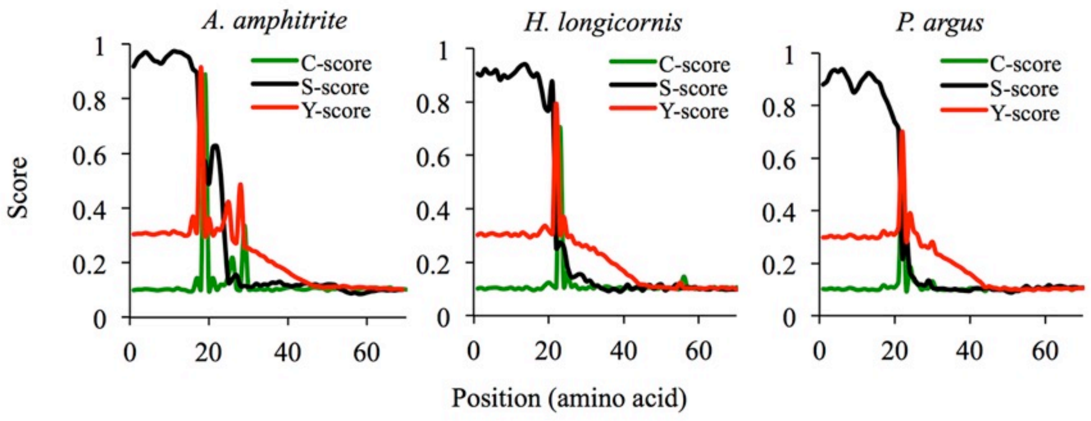

Figure S2. Signal peptide prediction of the CUB-serine protease from *A. amphitrite*, *H. longicornis* and *P. argus*. The C-score (raw cleavage site score), S-score (signal peptide score) and Y-score (combined cleavage site score) were calculated using SingalP 4.1 Server.

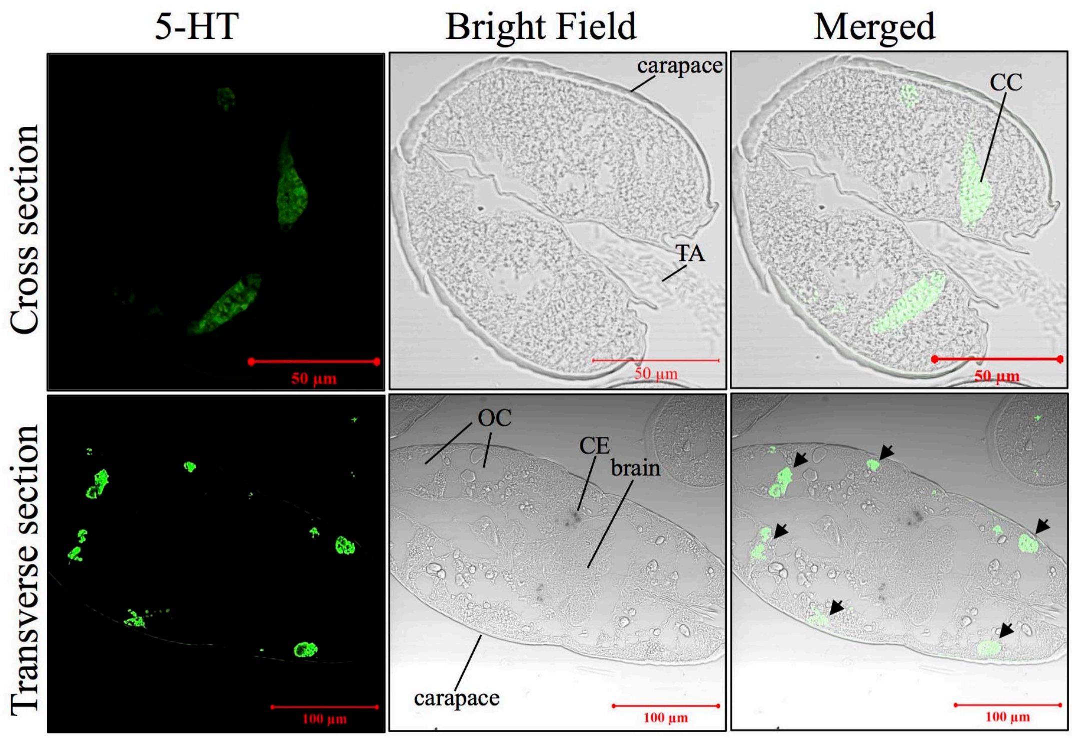

Figure S3. Immunostaining of serotonin in cyprid sections. Signals were revealed at the circumesophageal connectives in the cross sections. In the transverse sections, symmetrical singles were localized to nerve fibers nearby cyprid body wall (indicated with arrows). TA: thoracic appendage; CC: circumesophageal connective; OC: oil cell; CE: compound eye.

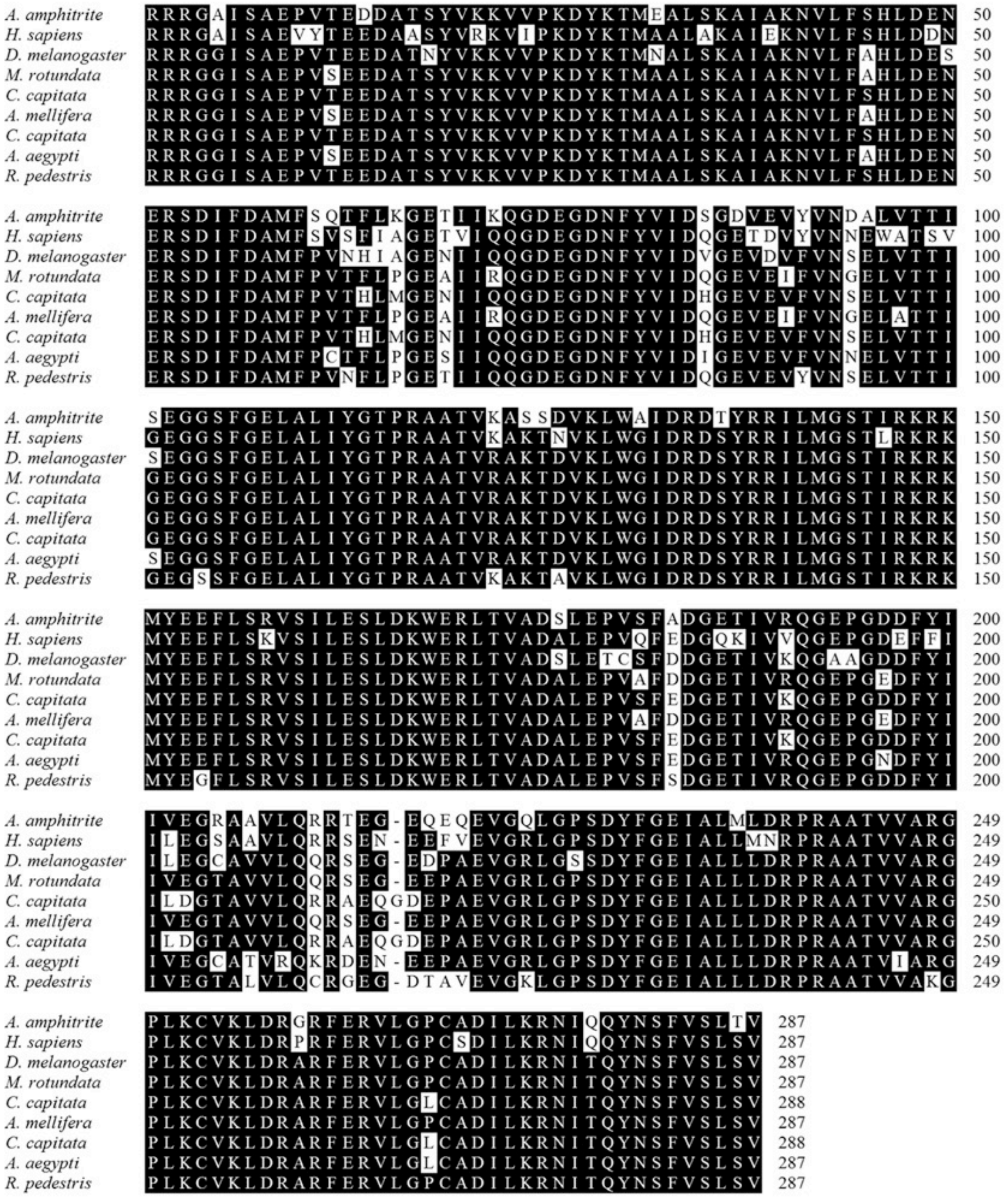

Figure S4. Amino acid alignment of deduced PKAα sequences. The sequences were obtained from *A. amphitrite*, *Homo sapiens* (NP002725.1), *Drosophila melanogaster* (NP524189.2), *Megachile rotundata* (XP003707654.1), *Ceratitis capitata* (XP004520213.1), *Apis mellifera* (XP006561792.1), *Ceratitis capitata* (XP004520214.1), *Aedes aegypti* (XP001661170.1) and *Riptortus pedestris* (BAN20503.1).

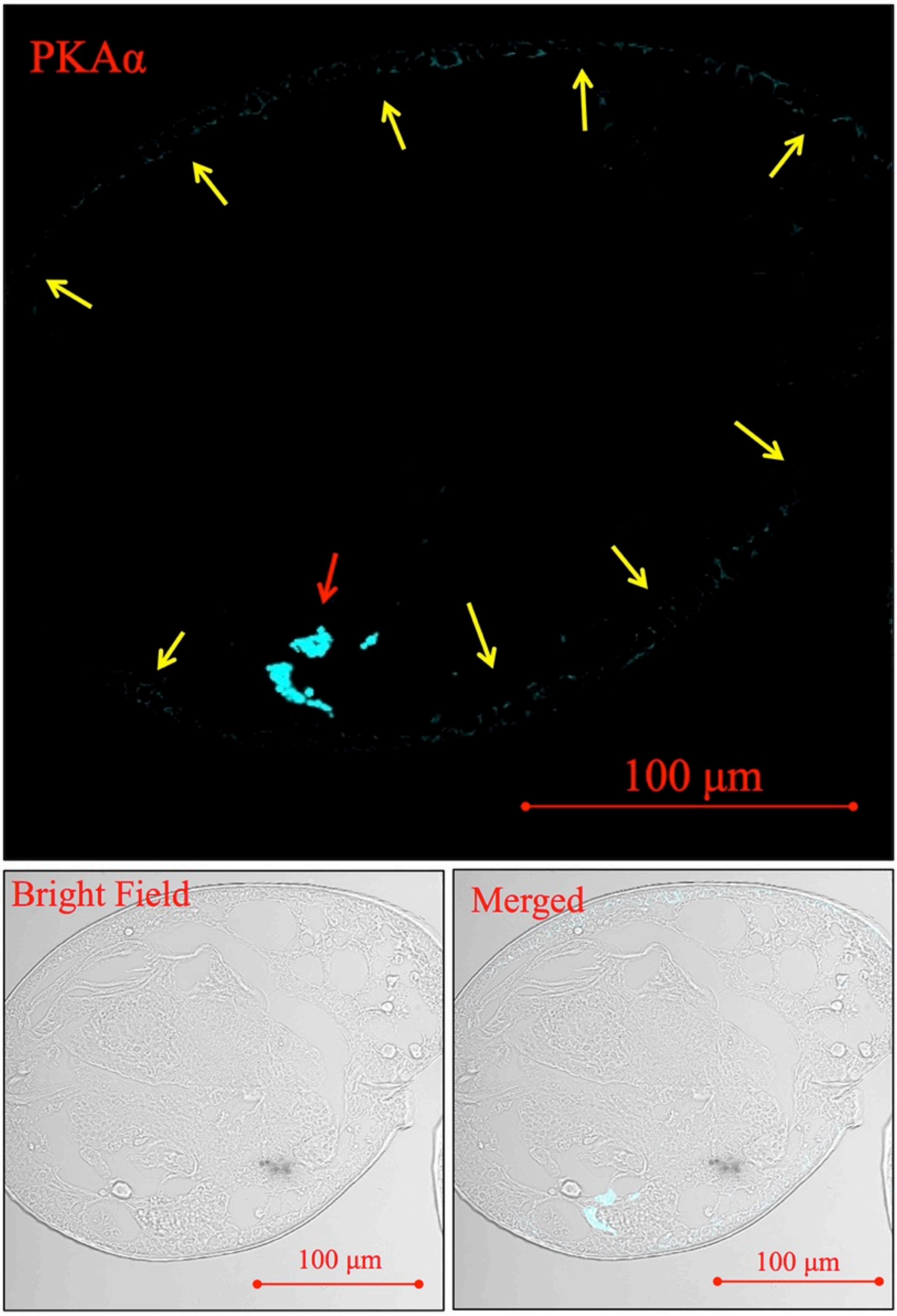

Figure S5. Immunohistology of PKAα revealed weak signals in the cells of the body wall. To better visualize the weak signal, the original red fluorescence (Alexa Fluor 594) is shown as light blue in the picture. The red arrow indicates the strong PKAα signal in the cement gland. Weak signals are shown by yellow arrows.

Table S1. Spectral counts of proteins that were significantly changed in response to SB203580 treatment. The spectral counts among three biological replicates are represented as the mean±SE.

No.	Contig	Protein name	Functional description	Control	SB203580	*P* value
**Energy production and conversion**						
1	Unigene23224	glycogen synthase	synthesize glycogen	9.3±3.1	24.7±3.1	0.0036
2	CL7578.Contig1	GG20479	NADH dehydrogenase (ubiquinone), produce ATP	5.0±1.0	8.7±1.2	0.0241
3	CL3129.Contig1	Mitochondrial 2-oxoglutarate/malate carrier protein (OGCP)	transport of 2-oxoglutarate across mitochondrial membrane	10.0±2.0	15.3±1.2	0.0161
4	CL6901.Contig1 Unigene11462	dihydrolipoyl dehydrogenase, mitochondrial-like	pyruvate decarboxylation	1.3±2.3	6.7±1.2	0.0232
5	CL1569.Contig1	V-type proton ATPase subunit B	pump protons across plasma membranes	79.3±6.1	62.0±7.2	0.0337
6	CL791.Contig1	Vacuolar ATP synthase catalytic subunit A	pump protons across plasma membranes	93.3±7.0	65.3±9.5	0.0146
7	CL593.Contig1 CL593.Contig2	AGAP006099-PA isoform 2	Ubiquinol-Cytochrome C Reductase, generation of ATP	36.0±6.0	54.7±5.0	0.0145
8	CL1.Contig245	Arginine kinase	maintennance of ATP level	150.0±21.4	102.0±7.8	0.0010
9	Unigene11511	Mitochondrial ATP synthase subunit g	Mitochondrial ATP synthase	5.3±2.3	0.7±1.2	0.0352
**Secondary metabolites biosynthesis, transport and catabolism**						
10	CL15192.Contig1	cytochrome P450 15A1	drug metabolism	14.0±5.3	2.7±1.2	0.0223
11	Unigene30671	cytochrome P450 2P3	drug metabolism	8.0±3.5	0.7±1.2	0.0254
12	CL7151.Contig1	cytochrome p450	drug metabolism	92.7±8.1	73.3±8.1	0.0428
**Cell cycle control, cell division, chromosome partitioning**						
13	CL9561.Contig1	GL10641	form the nuclear lamina	8.7±1.2	4.7±1.2	0.0132
14	CL2695.Contig1	Lamin-C	form the nuclear lamina	18.7±4.6	10.0±2.0	0.0406
**Amino acid transport and metabolism**						
15	CL15271.Contig1 Unigene5261	serine proteinase-like 2a	protease	16.7±2.3	5.3±3.1	0.0069
16	Unigene10435	saccharopine dehydrogenase	metabolism of the amino acid lysine	1.3±2.3	6.7±2.3	0.0474
17	CL11184.Contig1	sluggish A CG1417-PE isoform 2	conversion of proline to glutamate	1.3±1.2	8.7±3.1	0.0177
18	CL16496.Contig1	spermine synthase-like	convert spermidine into spermine	2.0±3.5	11.3±4.2	0.0405
19	CL374.Contig1	GI17616	serine-type endopeptidase activity	16.7±1.2	0.0±0.0	0.0000
20	CL6481.Contig1	GH20777	serine-type endopeptidase activity	10.0±4.0	0.0±0.0	0.0123
21	CL7.Contig19	CUB-serine protease	signal transduction	101.3±9.5	72.7±11.4	0.0284
22	CL1088.Contig2	serine protease	protease	14.0±5.3	2.7±3.1	0.0325
23	CL3.Contig27	GA26108	peptidease inhibitor	29.3±1.2	0.0±0.0	0.0000
24	CL3.Contig24 CL3.Contig30	GA26108	peptidease inhibitor	26.0±2.0	0.0±0.0	0.0000
**Nucleotide transport and metabolism**						
25	CL6274.Contig1	s-methyl-5'-thioadenosine phosphorylase-like	methionine metabolism	6.7±3.1	0.7±1.2	0.0335
**Carbohydrate transport and metabolism**						
26	CL17624.Contig1	glucose-6-phosphate 1-epimerase-like	isomerases	17.3±2.3	22.7±2.3	0.0474
27	CL11285.Contig1	Probable methylmalonate-semialdehyde dehydrogenase	generate acetyl-CoA	11.3±9.2	30.7±6.4	0.0409
28	CL4410.Contig1	Phosphotriesterase-related protein	hydrolase activtity on easter bonds	8.7±2.3	0.7±1.2	0.0058
29	CL992.Contig1	beta-N-acetylglucosaminidase	degradation of glucuronic acid-containing glycosaminoglycans	75.3±4.2	9.3±9.0	0.0003
30	CL304.Contig4 CL304.Contig5	epidermal retinol dehydrogenase 2	retinoic acid biosynthesis	7.3±1.2	16.0±3.5	0.0147
**Lipid transport and metabolism**						
31	CL17009.Contig1	methylmalonyl-CoA epimerase, mitochondrial	degradation of branched chain amino acids, odd chain-length fatty acids, and other metabolites	1.3±1.2	7.3±2.3	0.0158
32	CL4572.Contig1	propionyl CoA carboxylase, alpha polypeptide	fatty acid beta-oxidation	16.7±2.3	27.3±4.2	0.0178
**Translation, ribosomal structure and biogenesis**						
33	CL6243.Contig1	advillin-like	regenerative sensory axon growth	11.0±1.4	6.0±2.0	0.0249
34	CL6482.Contig1	transmembrane protein 2	multicellular organismal development	35.3±6.4	20.0±5.3	0.0332
35	CL11.Contig4	Polyadenylate-binding protein 1	cytoplasmic regulatory processes of mRNA metabolism	17.3±1.2	14.0±0.0	0.0075
36	CL14024.Contig1	60S ribosomal protein L34	ribosomal protein	2.7±3.1	11.3±3.1	0.0255
37	CL15766.Contig1 Unigene36236	S10e ribosomal protein	ribosomal protein	42.7±5.0	64.0±2.0	0.0024
**Cell wall/membrane/envelope biogenesis**						
38	CL4543.Contig1	peroxiredoxin 6 protein	regulation of phospholipid turnover	6.0±2.0	0.0±0.0	0.0065
39	CL2680.Contig1	Transmembrane protein 69	integral component of membrane	2.0±2.0	7.3±2.3	0.0390
**Cell motility**						
40	CL14396.Contig1 CL9899.Contig1 Unigene5067	neither inactivation nor afterpotential protein C	encode photoreceptors	15.3±2.3	4.0±3.5	0.0092

Table S1. Continued

No.	Contig	Protein name	Functional description	Control	SB203580	P value
**Posttranslational modification, protein turnover, chaperones**						
41	CL888.Contig1	Gasp protein	control of myostatin activity;preventing myostatin proteolysis	18.0±7.2	35.3±4.2	0.0226
42	CL6240.Contig1	60 kDa heat shock protein, mitochondrial-like	protein import and macromolecular assembly	6.7±3.1	22.0±3.5	0.0045
43	Unigene11234	AGAP009694-PA	protein folding	5.3±6.1	16.0±2.0	0.0453
44	CL2410.Contig1	14-3-3 protein epsilon-like	Adapter protein affecting various pathways	5.3±1.2	2.0±0.0	0.0075
45	CL8957.Contig1	thioredoxin-dependent peroxide reductase	protects radical-sensitive enzymes from oxidative damage	5.3±1.2	16.7±1.2	0.0003
46	CL2356.Contig1 CL4955.Contig1	glutathione S-transferase	detoxification	0.0±0.0	20.7±7.6	0.0091
47	CL4041.Contig1	glutamine:fructose-6-phosphate aminotransferase 1	regulating the availability of precursors for N- and O-linked glycosylation of proteins	0.0±0.0	5.3±3.1	0.0390
48	CL14928.Contig1 CL16649.Contig1	cathepsin D-1	lysosomal aspartyl protease	48.0±8.7	27.3±1.2	0.0152
49	CL1199.Contig2	glutathione reductase	resisting oxidative stress	26.0±3.5	10.7±7.0	0.0275
**Signal transduction mechanisms**						
50	CL3558.Contig1	cAMP-dependent protein kinase	regulation of glycogen, sugar, and lipid metabolism	14.7±1.2	10.0±2.0	0.0249
**Extracellular structures**						
51	CL3.Contig8 CL3.Contig20 CL3.Contig33	dumpy	gigantic extracellular protein	16.7±3.1	0.0±0.0	0.0007
52	CL3.Contig21	dumpy	gigantic extracellular protein	19.3±8.3	0.0±0.0	0.0158
53	CL336.Contig1 CL336.Contig5	cuticular protein	cuticular protein	30.0±3.5	11.3±1.2	0.0009
54	CL1239.Contig2 Unigene4984	CG42749, isoform C	cuticular protein	5.3±1.2	0.0±0.0	0.0013
55	CL888.Contig2	cuticular protein analogous to peritrophins 3-C5 isoform 1	cuticular protein	18.0±7.2	35.3±4.2	0.0226
56	CL21.Contig3	chitin binding protein	bind chitins	0.0±0.0	7.3±3.1	0.0142
57	CL14958.Contig1	AGAP011936-PA	chitin binding	0.0±0.0	10.7±2.3	0.0013
58	CL8680.Contig1	chorion peroxidase	shell formation	14.0±7.2	0.0±0.0	0.0282
**Cytoskeleton**						
59	Unigene25886	Myosin heavy chain CG17927-PF isoform 3	Moysin component	22.7±4.2	8.7±2.3	0.0070
60	Unigene15759	Plectin-1	link cytoskeletons and different cells	5.3±1.2	1.3±1.2	0.0132
61	CL7850.Contig1	f-actin-capping protein subunit alpha-like isoform 2	protect the growing ends of actin filaments	6.7±1.2	12.7±1.2	0.0031
62	CL2450.Contig1	CBN-CYK-1 protein	actin filament organization	18.7±5.0	4.0±4.0	0.0168
**Function unknown**						
63	CL2677.Contig1	HyTSRp1 protein	unknown	22.0±2.0	12.0±4.0	0.0179
64	CL2236.Contig1	CG15449-PA	unknown	6.7±2.3	1.3±1.2	0.0232
65	CL7397.Contig1 Unigene5246	GL15817	unknown	12.0±2.0	20.0±3.5	0.0257
66	CL9979.Contig1	AGAP005990-PA	unknown	12.0±0.0	8.7±1.2	0.0075
67	CL7745.Contig1	MGC147117 protein	unknown	6.7±3.1	15.3±2.3	0.0173
68	CL2357.Contig1	GA20276-PA	unknown	9.3±4.2	0.7±1.2	0.0255
69	CL2646.Contig1	Putative oxidoreductase yrbE	unknown	19.3±3.1	38.0±5.3	0.0061
70	Unigene34863	C9orf68 homolog	unknown	10.0±3.5	20.0±2.0	0.0123
71	CL74.Contig3 CL74.Contig5 CL74.Contig6	AGAP004418-PA	unknown	31.3±2.3	23.3±2.3	0.0132
72	CL14828.Contig1	AGAP006918-PA	unknown	3.3±3.1	9.3±1.2	0.0335
73	Unigene9033	unknown	unknown	0.0±0.0	9.3±1.2	0.0002
74	CL46.Contig1 Unigene11352	unknown	unknown	18.0±2.0	1.3±1.2	0.0002
75	CL1824.Contig3	unknown	unknown	27.3±5.0	4.0±4.0	0.0033
76	CL2298.Contig1	unknown	unknown	60.0±2.0	16.7±14.7	0.0073
77	CL1824.Contig2	unknown	unknown	18.0±4.0	5.3±3.1	0.0121
78	Unigene5401	unknown	unknown	184.7±16.2	144.0±5.3	0.0144
79	CL166.Contig1	unknown	unknown	109.3±16.0	76.7±7.6	0.0332
80	CL4124.Contig1	unknown	unknown	2.7±4.6	12.0±3.5	0.0488

Appendix I: Protein sequences of the contigs involved in the present study.

>Unigene23224_Ba_mix
GRWLVDGNPQIILFDVGSASWKMNEMKDELWNLAGIGIPNPDVECNDAVVFGYMVA
EFLQQFRNRLPEFTDGPPRMVAHFHEWLAGVGLILARLRKIDLATVFTTHATLLGRFL
CAGNTDFYNNLPYFNIDEEAGKRQIYHRYCMERASTHLCHTFTTVSEITGLEAEHLLK
RKPDIVTPNGLNVKKFSALHEFQNLHALSKAKINDFVRGHFYGHFDFNLDKTLYFFIA
GRYEFGNKGADIFIESLSRLNHYLKSSGSDVTVVAFMIFPTKTNNFNVESLRGHAVTKT
LKDSIEDIQKKIGSRMFEICMTGRLPESTELLTREDNVRLKRCIYSMQRSCLPPITTHNV
VNDGEDPVLRSLRRCHLFNDKSDRVKVVFHPEFLSHTNPLLGMDYEEFVRGCHMGVF
PSYYEPWGYTPAECTVMGIPSMTTNLSGFGCFMSEHIADPMSYGIYIVDRRSIGLEDSIR
ELAKYMYDFTRLNRRQRIIQRNRTERLSDLLDWRNLGIYYRRARQLALHAVYPEMAV
SEEHGGRMSYPRPLSEPPSPTTSRSTTPAPSEIGSDDEVDDEEELLELAIKD

>CL7578.Contig1_Ba_mix
ISPQEKRLIEERRNHRMKMKAQWQKLVSDPHSHKHGGYVFDPAMQRFMSMRVTQY
ESFRPTLGNFFRGIGITAIPILLFAQLMHWDRTRKEKEFSTGQVAYKDRLWK

>CL3129.Contig1_Ba_mix
QETSIPNYVKFIFGGSAGMAATMFVQPLDLLKNRMQMSGMGGAAREHKTSFHFARAI
VMKEGVRGMYSGLSAGLLRQATYTTTRLGIYTELFERASKDGKPPPFFTKAAIAMVAG
LVGSFIGTPAELCLIRMTSDGRLPEAERRNYKHAFDALFRVAREEGVTALWRGAVPT
MGRAVVVNAAQLVSYSQAKQSLMSTGYFSDNIFLHFCSSMISGLVTTAASMPVDIAKT
RIQNMKVVNGVPEYRGGLDVLAKVVRNEGVFALWKGFTPYYARIGPHTVLTFIFLEQ
MNKAYKKFVLHDTSAGG

>CL6901.Contig1_Ba_mix
DGSKEVIKTKNILIATGSEVTPFPGIEIDEDTIVSSTGALSLKQVPKKLVLIGAGVIGLELG
SVWSRLGAEVTAVEFLGHVGGVGIDAEVSKNFQRILGKQGIKFKLGHKVTGAERSGDN
ISVSVENVKDSSKKETLDCDALLVCVGRRPYTDRLGLEKMGIERDEKGRIPVNSRFQTV
IPNIHAIGDCIHGPMLAHKAEDEGIVCVEGIAGGPVHIDYNCVPSVIYTFPEVAWVGKT
EEDLKADGVEYKVGKFPFSANSRAKTNQETDGFVKVLGDSATDRMLGVHIIGPGAGE
MINEAVLAMEYGASCEDLARVCHAHPTCSEALREANLAAYCGKAIN

>Unigene11462_Ba_mix
DLVVIGSGPGGYVAAIKAAQLGLKTVCVEKNETLGGTCLNVGCIPSKALLHNSHLYHEA
VHEFKNRGIDVEGVKLNLEQMMNTKKKAVSGLTSGVAYLFKNNKVTHVKGHGTITGT
NEVTATAADGSKEVIKTKNILIATGSEVTPFPGIEIDEDTIVSSTGALSLKQVPKKLVLIG
AGVIGLELGSVWSRLGAEVTAVEFLGHVGGVGIDAEVSKNFQRILGKQGIKFKLGHKVT
GAERSGDNISVSVENVKDSSKKETLDCDALLVCVGRRPYTDRLGLEKMGIERDEKGRIP
VNSRFQTVIPNIHAIGDCIHGPMLAHKAEDEGIVCVEGIAGGPVHIDYNCVPSVIYTFPE
VAWVGKTEEDLKADGVEYKVGKFPFSANSRAKTNQETDGFVKVLGDSATDRMLGVH
IIGPGAGEMINEAVLAMEYGASCEDLARVCHAHPTCSEALREANLAAYCGKAIN

>CL1569.Contig1_Ba_mix
MPTTLTPHQARTEHTMAITRDYISQPRITYKTVSGVNGPLVILEGVKFPKFGEIVNLTL
GDGTDRQGQVLEVSGSKAIVQVFEGTSGIDAKNTICEFTGDILRTPVSEDMLGRVFNGS
GKPIDKGPPVLAEDFLDIQGQPINPWSRIYPEEMIQTGISAIDVMNSIARGQKIPIFSAAG
LPHNEIAAQICRQGGLVKLPTKSVMDDHSDNFAIVFAAMGVNMEAARFFKQDFEENG
SMENVCLFLNLANDPTIERIITPRLALTTAEFLAYQCEKHVLVILTDMTSYAEALREVS
AAREEVPGRRGFPGYMYTDLATIYERAGRVEGRNGSITQIPILTMPNDDITHPIPDLTG
YITEGQIYVDRQLHNRQIYPPINVLPSLSRLMKSAIGEGMTRKDHADVSNQLYACYAIG
KDVQAMKAVVGEEALTADDLLYLEFLTKFEKHFITQGNYENRSVFESLDIGWQLLRIF
PKEMLKRIPASTIAEFYPRENK

>CL791.Contig1_Ba_mix
GCGERGNEMSEVLRDFPELTVEIGGQTESIMKRTALVANTSNMPVAAREASIYTGITLS
EYFRDMGYNVSMMADSTSRWAEALREISGRLAEMPADSGYPAYLGARLASFYERAGR
VNCLGNPNREGSVSIVGAVSPPGGDFSDPVTSATLGIVQVFWGLDKKLAQRKHFPSIN
WLISYSKYIRALDDFYEKNFQEFTALRTKVKEILQEEEDLSEIVQLVGKASLAETDKITL
EVAKLLKDDFLQQNGYTPYDRFCPFYKTVGMLKNMIAFYELARHAVESTAQADNKIT
WNVIRETMSQTMYQLSSMKFKDPVKDGEAKIKADFEALHEEMQQAFRNLED

>CL593.Contig1_Ba_mix
MASKLMKSNILRSLPARRFAAQAATKSSDYGVLPREPLKTTTLPNGVVVASIENQSPLS
RVAIAVRGGSRYETPDNRGATHLLRIVAGLTNAENTAFGLTRELQQLGASLACSADRE
VLIYSADATRNHMDVVMELLANAATRQVFKPWEITDIKPHLKLDVETTPAEVSLIEDL
HLAAFRQSDLGNSVVAPHHLIGQLGPDQLASHLSRTLLAGRTAVCGTGIDHETLVEYA
QGLQLPQGEGPAPAARYGGGELRTEIGGGVALVAVAAEGAGRSSATAAQQAVLQAVLG
AGAVVKYGAGRGVLGAAAAQAGGAAAALNAVYSDTGLVGYTVAAEADKAGQVIEAVN
KAFKTTSLTAEDVNRGKALAKAAVLRADDNSGSCVETMGVQGASLGKVSAVSDALAA
IDAVKLSDVQTLHKRLVGGKLSMAARGNLRTVPYLDQL

>CL593.Contig2_Ba_mix
MASKLMKSNILRSLPARRFAAQAATKSSDYGVLPREPLKTTTLPNGVVVASIENQSPLS
RVAIAVRGGSRYETPDNRGATHLLRIVAGLTNAENTAFGLTRELQQLGASLACSADRE
VLIYSADATRNHMDVVMELLANAATRQVFKPWEITDIKPHLKLDVETTPAEVSLIEDL
HLAAFRQSDLGNSVVAPHHLIGQLGPDQLASHLSRTLLAGRTAVCGTGIDHETLVEYA
QGLQLPQGEGPAPAARYGGGELRTEIGGGVALVAVAAEGAGRSSATAAQQAVLQAVLG
AGAVVKYGAGRGVLGAAAAQAGGAAAALNAVYSDTGLVGYTVAAEADKAGQVIEAVN
KAFKTTSLTAEDVNRGKALAKAAVLRADDNSGSCVETMGVQGASLGKVSAVSDALAA
IDAVKLSDVQTLHKRLVGGKLSMAARGNLRTVPYLDQL

>CL1.Contig245_Ba_mix
SQPKDSKPAAKSSNMVDAATMQKLDAGFQKLQAATDCKSLLKKYLTKEVYDSLKNKK
TSFGSTLLDVIQSGVENLDSGVGVYAPDDEAYRVFADLFDPIIEDYHGGFKKTDKHPNK
DFGDVSTVGNVDPEGQFVISTRVRCGRSMKGYPFNPCLTEAQYKEMEDKVSSTLSGLD
GELKGTFYPLTGMAKDVQQKLIDDHFLFKEGDRFLQAANACRFWPTGRGIYHNNDKT

FLVWCNEEDHLRIISMQMGGDLGQVYRRLVNAATDIEKRIPFSHHDRLGFLTFCPTNL
GTTIRASVHIKVPKLAADKKKLEEVAAKYNLQVRGTRGEHTEAEGGIYDISNKRRMGL
TEYEAVKEMHDGILELIKIEKSL

>Unigene11511_Ba_mix
MAGVVAKLPAMGTNLVNAARPRLATFLKYARVELTPPSPADVPAIQQGLSNLVKSAR
TGAWKNLTVKEATVNALVGAEVVFWFFIGECIGKGSLVGYQV

>CL15192.Contig1_Ba_mix
RPTNYPPGPPMFLIFGNLFETFRSLKGDFEVMVKEHRKKYGDIMAYIMPGNFKIVHLL
NYDLIKESCARQEFSGRPSNFIVLYRSFQKKLGVILNDGPAWQEHRRFTLRHLRDLGFG
TNSMETIILEEFEHLAKEMEQDMHSPMKVNQRFNLTVLNVLWRLVASKRLDRSEPEA
KQRVKEVNDFVTITGPSNPLSMFPFLRFISPNSFGLKELKNNRDNSLAMFQELMKEHR
QTLDRAAPRDFIDRFLIEMESPDAAARSFTDENLSIVCMDLFLAGMETTSTSLTWALL
LLVMHPDVQTRVQQEIDAVLGVGADRRLPSYTDRANMPYTEATLQEVSRRATVVPRA
VGHSALQDAPLGGYTVAKGSLVLMHLDVVHMDPAYWGDPDTFRPERFINADGTV

>Unigene30671_Ba_mix
FGIGRRFCLGESLARMEMFVLFTCLLQRFRFSAAPGQTLSLESRVAAVRQPNEF

>CL7151.Contig1_Ba_mix
VVFFVVLLVTYLWVTRPKRPPNFPPGPPTFLIFGNVIDLVRTASNDFQKHLNRLRSEH
KSDIIGMVDPFGQPLVYVFGGELVKEAGNITELTGRPELFTVMYLSEMKRAGIFFNEGP
LWQEQRRFALKHLRDLGMGKSVLNTMIADEYALFAEHLKKVVGQEVKANNLFITVVL
NIIWKMVASRRFDYDDPERQRLQDIVTEFTQITGPQNILFLFPSARFVAPESSGFNKIK
VYHSAMQGLFGPCIAEHRATLDPSHPRDFIDAFLIESQRPDAEKRGFDENNLRTLCLD
FFIAGSETTSLSMTWALLLMVLYPDVQAKVQAELDEVVGEGRLPSYEDRPRLPYTEAV
MTEIWRYTSFVPLAVPHRSSVGPAQLGGYTLPKDTTVMIHLQSAHHDRGHWGDPDV
FRPERFIGEDGKFRKDDYFVPFGLGKRACLGESMARTEFFTFLSCMLHQFHVRLPKGA
KKPS

>CL9561.Contig1_Ba_mix
RTTMSTSSGGSAATSPVRARSPHSPTRTSRLQEKEQLAGLNDRLAAYIEKVRNLELEN
SRLTHQVRSSQEVVTREVSNIKSLYESELADARRLLDETAKERAKLQIDLGNLKTERD
DLQSRLIRRERELDNAEKKISMQDMQIRDLQGRLHQALQDKKKAQEELEEVQLENMK
LKKQLDEVRKQLESEALGRVDMENRCQSLKEELEFKTNVYEGQLTETRAKKTEEITEL
DGRLQQEYEVRLRESLQDLRDQYEAQMKANREEIEAIYNLKMDELRNSQARNQLSTA
TTLKELREMRSRIEGLTGKI

>CL2695.Contig1_Ba_mix
SARMFKKTTSTMSVSSGGSAAASPARSRSPSSPARTTRLEEKQQLAGLNDRLAAYIEK
VRSLEAENNRLSLQVRTSQEVVTREVSNIKSLYESELADARRLLDETARERAKLQIDLG
NLKTERDDLAAKLVRRERELEGAEKQVSLQEAQVRDLQGRLNQALADKKKAQDDLK
DAQLENMKLKKQLDEVRKQLESEALARVDMENRCQSLKEELEFKTNVYEEQLTETRS

KKTEEITELDGQLQSEYEARLRESLHELREQYEMQMRVNREEIETIYESKLEDLRAAQS
RNQSSTATTLRELRETKARIDDLLGKISDLEGTNTTLLARVRDLERQLDDERTSSASM
LVAKNEEIERLQELMQQQLQEYQDLMDIKVALDVEIAAYRKLLESEEARLNLSPGGRH
VVEVSASSSARGSRATPSAGRAGKRKRMVLESEEASTSGFAVANTCHGDVEIVDECAD
GKFVKLRNKSNKEVSLGGMQLVRKADGNETDYKFHRAIKLGAGEETTVWASEAGAV
HDAPHSLVMKGQTWFVADKMTTTLYDMEGEEIAQRVSQRENRSASFTTSRQQVIAT
GSEE

>CL15271.Contig1_Ba_mix
GACVPYYLCKDGDIVTDGAGLIDMRFGGDDEDSKSNSQCPNFLDVFCRVPLTEPDPDI
LGELTEYEYQPSCGHRNPEGVSIILDGFKEGESQFGEFPWMGILLEDELLDYDQVIQRY
VCGCSLIHPQVIMTGAHCINGKDPAKLTVRLGDWDTQNDKEIFPHEHHRVVDMEIHE
RFNSANLRYDVGLLFLEKPVVLQQHIDTICLPAANEDFDLASCVATGFGKDQFHGGRF
QNIMKQVDLPIVPHRTCQNRLRETRLGRWFRLHRTFTCAGGEAGVDTCRGDGGSPLA
CPSKEDPSKYVQVGIVAWGIGCGEDGVPGVYASVPKLVGWVNEKMAEHGIYTDLQV

>Unigene5261_Ba_mix
GACVPYYLCKDGDIVTDGAGLIDMRFGGDDEDSKSNSQCPNFLDVFCRVPLTEPDPDI
LGELTEYEYQPSCGHRNPEGVSIILDGFKEGESQFGEFPWMGILLEDELLDYDQVIQRY
VCGCSLIHPQVIMTGAHCINGKDPAKLTVRLGDWDTQNDKEIFPHEHHRVVDMEIHE
RFNSANLRYDVGLLFLEKPVVLQQHIDTICLPAANEDFDLASCVATGFGKDQFHGGRF
QNIMKQVDLPIVPHRTCQNRLRETRLGRWFRLHRTFTCAGGEAGVDTCRGDGGSPLA
CPSKEDPSKYVQVGIVAWGINCHQAGVPGVYADVVALMPWIRS

>Unigene10435_Ba_mix
RAYPMQAYINAGAQIKEDIGEASVIMGVKQVPIDALIPNKTYCFFSHTIKAQEANMPLL
DAILEKNIRLLDYERMCDERGQRVVAFGKYAGVAGMINIMHGMGLRLLALGHHTPFM
HIGPAHNYRNSEMARQAVRDAGYEIALGMLPRSLGALTFVFTG

>CL11184.Contig1_Ba_mix
ADAAKLGPQRDQLDLTFTDTRAAFQSKTTWEVFRGVLVYTLCTSRYLVEHNATLMKL
FRKLLGQKLFHTVMRATFYGQFVAGADQASIRPQIENMRSFGVKSILDYSVEEDISTEE
AEQAEMRSCSTPGEQEQELTVQKDPMARYHAHREFADRRVKVQSARTYFYMNEAQC
EKNMETFLKSIEAVSGSTQCTGLAAIKMTALGRPQLLMQLSEVIMRTRRYLAELAGSE
RATDVISQDVSVEQIQSRLKETSGPGVEQWLKGLTYDQDGLIHLFSWSGLIDTKTLMS
DLFRVPNLTTGRMEPLITSLTKDEEEMFKNMMRRLHTIFSYARECDVRVMVDAEQTY
FQPAISRLTMELMRKYNREKAIVFNTYQCYLKEAYHNLSVDLDQADRQHFYFGAKLV
RGAYMEQERYRAASLGYEDPINPTFEATSEMYERCLLETLRRIQHYKKYGQPRKLAIM
VASHNEDTVRFTVQKMDELDIKREDKVVCFGQLLGMCDHVSFPLGQAGYSVYKYVPY
GPIDEVLPYLSRRAQENKGILTKVAKERKMLRSELLRRLVSGQLFYKPVGNYTPV

>CL16496.Contig1_Ba_mix
MSFNTTLLDFSVAPEIITNPVASATLHQQVEEVVGAVVPGLTRLIEDLLPSGGFHMTYT
AENETFVTFRGHRSGLVSVQIENYGETFLMENRAVVELEKRIRAKIGAHRGKAILPLKR

GRDIDSYFYTSDERLLEYGVLETIFDEVTPYQHVQVVRTRDFGKILVLDGFHNMSEKDF
IYTETLMGGENYEGKTALILGGGDGGLLCELLKQNPKFVTMVEIDEVVIRECRKHLRAI
CGDCLDKFDGDNYKIIVGDAFQYLKDSVQEGRTFDYIFYDLSDVPVSPRHDRDLWNM
VKDVLQDSMSLLPAGGKYMSHVMGVNCPEALEEFERLVDELPAKTSRTSSSAYVPSF
METWVFYQVT

>CL374.Contig1_Ba_mix
GSTCDTPDGRSGVCLARSSCRWTRESSEDDFNSLVRTLGACVSTDSFSPRGEPSVRGVC
CPGQGAASAPATTTARPPGGTQWWQTTWSPPPSATTTTTTTRRPATVPWWQQQQP
AASSQRTEPAGTTLHPPYITHAWQATRGSTSRPPAAPTHPWQKPPGSTTVQSPTTTA
WWATTTTTRGTTTTRRPTTTAWWATTATTKKPTTTAWWATTATTKGTTTTKRPT
TTAWWATTTKSTTAAAPPASGGGGPYSCGTRDPEGSDRIVGGKQAVQGEFPWLAGIF
KNGRQFCGGSLIDEKHILTAAHCVAHMSSYDVSQLLIRLGDYDVSTPFEAKHETYKVA
RVVRHKGFSEKTLHTDIALITLSKPVKYKSNIRPICLDSGVSRGAAGQTVTVAGWGSLR
EGGRQASIMQKVSLKVWDNSKCRSTYGPSAPGGIISSMLCAGRQGKDSCSGDSGGPLM
HISGVVYQVGVVSWGIGCGKAQYPGVYTRVSEMRDWIDRN

>CL6481.Contig1_Ba_mix
CGKTKFSTRIVGGTEPPAHAFPWLVALQTRKRKKQFCGGTIINERFILTAAHCVDRRK
PTGLAIAVAKHKRRDDPTEKIFNVKNIKIHEKYNRATQDRDIAIIELTRSLRSMIADER
GWVRPICLPRKTCRSENNCYTARSAILAGWGLLDSTFFEGPATVQYVKVPVLSNNQCQ
ENYAKDRIGITRRMVCAGYPDGGKDSCQGDSGGPMAVVSADEVYTLAGVVSFGKGCA
LPGYPGVYVRVSEFTDWIRDNTR

>CL7.Contig19_Ba_mix
TDGDGDYSNNVDCYYQLYADQAGDQLSVTCSQFDVESHSSCAYDWLSVDGNKFCGSN
GPSGVAAIDQMAIHWHTDYSVVRSGFVCAVTVGEAPPPTTTAAPPPPSDNCACGNVN
RASRIVGGVQTEANEYPWQAGLVSTGGSRTWCGGTVINSRYVLTAAHCTAGSSASRIQ
VLLREHLINQDDGEIRFNIAQIKNHPNYNSGTYANDFSLLKLATAITFPADNKIAPACL
PTASNDFVGADAIVTGFGTTASGGSQATALREVTVPVISNSACQSAYSGYSITSSMVCA
GLLGTGGKDSCQGDSGGPLVSLENNRYSLIGVVSWGISCADPSYPGVYARVTDALTWIS
TNAAD

>CL1088.Contig2_Ba_mix
RPDPPSTSREVTCQASQECVRPDLCDENGRIITDGSGLLNARFNNREFCRTPGGGKGR
CCLPADPEPQPLVCGQSLDIRILGVTPDSTEANFGELPWQAIISYTNYTFICGGSLVGRQ
HVVTAAHCVQSLQPGAIQVRLGEYRVNAEDEPLPHQDQGVSEIHIHPQYERGPLFYDL
AVLRLSSPVQLDSHIQPICLPTPGQQFTGRCVASGWGKDAFEEGRFSTILKKVRLPMVS
HEQCQENMRNTRLGSFFVLNKSFVCAGGEEGVDACKGDGGGPLACPSNGRYTLAGVT
SWGIGCGARDVPGIYASAEAGLDFIRQHADS

>CL3.Contig27_Ba_mix
CPAGQECVQTRSGRVCRPRDDSCRPKMSPGGCPGGSYCGQEGRCTPLLDVDAPCWSG
VQCLHHVCRGHVCKLDACPQLDSDQDCPEGQFCSSSQRGNICINRRADGEACSSDAQC

QSYRCDGRRCSRDTCKTLLSRDGCPDGHICQQGQHGNLCVPLLARGASCSSDRSCRSD
SCRAGRCYEDLCPTPLDTAGCDFSLLCVHTLIGNECMTKLTIGADCTADLQCLSNFCDN
NQCRRPLAKNGDSCRFDNDCFSRNCFKGICRPSLASNGERCQFDDECFSGNCFRGRVA
HREQ

>CL3.Contig24_Ba_mix
PGECQTPYSQSGCPYDQVCTADYRCVPLKPTGSRCRSEFECLSGKCRESRCVHDECLGE
GFNSGCPAGQECVQTRSGLVCRPRDNSCRPAMSAGSCPSGNYCGQEGRCTALLDVNAP
CWNDVQCLHHVCRGHVCKMDACPQLDSDQDCPEGQFCSSSQRGNICIDRRPDGEACS
SDAQCQSYRCDGRRCSRDTCKTLLSRDGCPDGHICQQGQHGKLCVPLLARGASCSSGR
SCLSDSCRAGRCYEDLCP

>CL3.Contig30_Ba_mix
PGECQTPYSQSGCPYDQVCTADYRCVPLKPTGSRCRSEFECLSGKCRESRCVHDECLGE
GFNSGCPAGQECVQTRSGLVCRPRDNSCRPAMSAGSCPSGNYCGQEGRCTALLDVNAP
CWNDVQCLHHVCRGHVCKMDACPQLDSDQDCPEGQFCSSSQRGNICIDRRPDGEACS
SDAQCQSYRCDGRRCSRDTCKTLLSRDGCPDGHICQQGQHGKLCVPLLARGASCSSGR
SCLSDSCRAGRCYEDLCP

>CL6274.Contig1_Ba_mix
IGIIGGSGFSDPSALGVEDAKDVEYDTPFGKPSSPLLTGTLCGVPVAVLARHGRRHTILP
SAVNYRANVYALRRHGCTHVLVSTACGSLREEYRPGQLVLLDQFIDRTTKRPLTFFDG
SCDEFVGICHLPMADPFCGETRGVLGEAAKELGITMHDKGTIVTIEGSRFSTRAESHMF
RSWGCDVINMTTVPEVTLGQGGG

>CL17624.Contig1_Ba_mix
MAAPTAVVVLDRGNNTTCTVNLHGATVVSWRVNNQEQLFVSKQAVFDGKKAIRGGI
PIVFPHFGPWSLGPQHGFARTSRWHLEKAPERLPSGDIEAIFVLMDSEYTHQFWKHSF
RLTYRLILREKELHFNIGVYNPLGSQDTFSFTLLLHTYFKCPDVRKCQVTGMHGCTFID
KTRDGAVYQEGRDVVMINEWTDRIYQNTPQEHIITNVVSGRKMRIQKFNLPDTVVW
NPWEHKAKEIADFGDEEFPNMICVEAGNVSTGIILPPNTVFEASQILQVM

>CL11285.Contig1_Ba_mix
LRLGQRFASVDTTKLFINGKFVESASSDWIDLHNPATNEVITRVPKSTPGEMQAAVDA
AKTAFPTWSRTSVLTRQQIMFNFQNLIKKNMKVLAANITSEQGKTLADAEGDVLRGL
QVVEHCCSITSLQLGETMSDVSRDMDTLSFRIPLGVTAGITPFNFPAMIPLWMFPTAV
VCGNTSVIKPSERDPGACMLLVQLLQEAGLPDGVVNVIHGEHEAVNFICDSPDIRAISF
VGSDQAGKHIYERGSRNGKRVQCNMGAKNHGVIMPDANKENMLNQLVGAAFGAAG
QRCMALSTAVLVGEAKHWLPELVERSSRLRVSAGTEPDADLGPVISPKAKERIIQLVES
GIREGAKCLLDGRAVKVPGYEHGNFVGPTILADVKPEMECYREEIFGPVLCVLTVDTLD
DAIELINNNPYGNGTAIFTTSGATARRFTHDIDVGQVGVNVPIPVPLPMFSFTGSRGSF
LGDANFYGKAGINFYTQLKTVTQLWRADDVTHSKAATTMPVMR

>CL4410.Contig1_Ba_mix

MATTKGKVQTVLGLVEPSSLGVTMMHEHMHLDFNVAFIPPEGKYAHKADCDFTLEN
SGWIRQNPYASRQNVTFCDAAVDAAMRDELRTYRENGGGAVVEVTNYGIGRDAALLA
AYSRDTAVTIVTGTGFYQHMFQLESDLALTVEEMHDRALKDITDGCPEAPLVKAGVIG
EVGSGWPIHDFERRAIQAAALGQQATGCPVNFHPGRDPAAAPEVLRIFQEAGGDVSNC
IMSHLDRSIQTAEGLSEFAKIGSYLEFDLFGIETSHYTLAPHVDMPSDAQRIQRIKHLVE
DGYEDRVVISHDIHTKHRLTKFGGHGFSHILENVVPMMKVRGLTEETINKILSTNPQA
AMTFK

>CL992.Contig1_Ba_mix
LTFPWMWECREGACLRTPVSEATNGMTLGGCKLTCGKGLTLWPKPTGTFTLGKTPV
SFKYSQVIVIVDAPQDVRGHVEKAGNRLKETLRDMRPPGGSCLELSSASDANLATRQL
TIRVTVEQSDASLTLKTDESYKLNITHTDADQLEAHIEAKTFFGARHGMETLSQLVAY
DDANDALMTVTADVRDAPVYPVRALTLDTSRNFISVAGLRRTIGAMAMNKLNTLHW
HITDTHSFPIEITGEPRLAQYGAYSPREVYTRAEVEELVQFALERGVRLLPELDMPAHV
GYGWQWGEKAGMGNLTVCLGKEPWQQFCVEPPCGQMNIINENLYKVLKTILVEYND
VFQPDLFHIGGDEINLNCWKTTPEIAAFVKEKGGDPTDDEEYMKLWIQFQERVYDM
WTSIAGEQKTILWTSHLTQRPAEESRLDPSKYIIQIWTTGKDQQIGDLLKQGYELILSN
YDAWYLDCGGSAWVGKGNNWCSPYKSWQTVYENSPRKIAQSFQSNSDAAASQPASQ
SDFSAQILGGSAALWTEQADDANLDNKLWPRGAALAERLWAEPETDSLAAQWRLVH
HRRRMVARGVHAETMQPKYCHQYDGTCYI

>CL304.Contig4_Ba_mix
METVLNGLLCVAQALFLWLQSVVLFFVPAKYREKSVSGDVALITGAGSGIGRLLALRLA
KRGARIVTWDVNAEGNEETVRQIRAAGGEAHAYTCDLSNREVIYKTAERVKREVGKV
TILVNNAGMVTGKPLLDIPDNLIEKTFEVNTLAHFWTCKAFLPDMMIAKKGHVVNIAS
LAGLQGMGRLTDYCASKFAAVGFHESLTVELKVGGHEGVKTTVICPFFINTGMFDGAK
SNIIPFLEPEYVAERIEQAILLNEEQVLIPGYLAALFYLKLAMPWRAYWTLGLAFKVER
AMDTFTGRTKQ

>CL304.Contig5_Ba_mix
METVLNGLLCVAQALFLWLQSVVLFFVPAKYREKSVSGDVALITGAGSGIGRLLALRLA
KRGARIVTWDVNAEGNEETVRQIRAAGGEAHAYTCDLSNREVIYKTAERVKREVGKV
TILVNNAGMVTGKPLLDIPDNLIEKTFEVNTLAHFWTCKAFLPDMMIAKKGHVVNIAS
LAGLQGMGRLTDYCASKFAAVGFHESLTVELKVGGHEGVKTTVICPFFINTGMFDGAK
SNIIPFLEPEYVAERIEQAILLNEEQVLIPGYLAALFYLKLAMPWRAYWTLGLAFKVER
AMDTFTGRTKQ

>CL17009.Contig1_Ba_mix
DSGVPKALWKLGRLNHVAIATPDLKKSTALFRDVLGADVSEPEALPEHGVRTVFINLG
NTKLELLHPLGPDSPITNFLDKNPSGGMHHICIEVDNIAEAVASLQQKQIRCLNEPRPG
AHGKPVVFLHPKDCAGVLVELEEA

>CL4572.Contig1_Ba_mix
DKIQSKKLAIDAGVNTIPGFEGEIRDVDHCVAVSREIGYPVMIKASAGGGGKGMRIARN

DDEAREGFRLSQQEAASSFGDDRILVEKFVDCPRHIEIQVLADQHNNAVYLNERECSIQ
RRNQKVIEEAPSVFLDEETRRAMGQQAVALARRINYSSAGTVEFLVDSARNFYFLEMN
TRLQVEHPITECITGVDLVQQMIRVAKGHKLKLKQEDIPIRGWAIESRVYAEDPFKNFG
LPSVGRLSKYIEPNHLPGVRCDSGVAEGSDISIYYDPMICKLVAYGNSRQEAIARSLDAL
DSYVIQGVTHNIPLLRDILTEPRFVSGDITTNYLPEVYPDGFQGHRLEGESLLRLAALSA
VIHVKHTQRSSLVSGHHALQPVATKWDLVV

>CL6243.Contig1_Ba_mix
GDVFVLDAREAVYVWTGKEANHMEKIQAAKTAQSIKDDHGGADIIVVESGKENEMSK
NEKALFNKLLPLSDRSVRPASAAPSDDKQERRVAEDIRLYRCSDEDGALKVTEVKGGP
LLQSDLDKNDTFIIDNGEQGIWVWVGRRATHKERAEAMRNAQGFIKKKNYSQATPVT
RVIDGGEPTDFKSLFQSWRDKDATTGLGKKATIGQVAKTIQTSFDASTLHEHPDLAAH
TQMVDDGTGKKEIWRVEDFDLKPVPTDKYGKFFSGDCYVILYTYHARGREEQIIYYWL
GSHSAQDEQGTAALKTVELDDSLGGRPVQVRVVQGKEPPHFLAMFGGRMVVYSGGVS
SAFESAEGVNGGHNATVGDTYLLQVRGTTNYNTHAVQVDCRAGSLNSNDCFVLRHGG
EVFVWCGKGATGDEREMAKTIAAEMHSEPQIIYEGQERDDFWAKLGGLEPYASSPRL
AEADQMQPARLFQCSNASGRFLVEEIPDFIQDDLVPSDVMLLDAWNTIFIWIGSGSNK
TERERAEKTAIEYLRTDPAGRGVGTPIVRVKQGCEPPTFTGFFGVWDDGLWESQPDW
EAVRRDVSAGNKGISEVTVAAAKAVELPVYELETLRQHDPELLPEDVDPSIKERYLAPE
VFEELFGMSRDAFSALPTWKQQQLKKKVDLF

>CL6482.Contig1_Ba_mix
CPHEGTLKSWSDAASWASGTVPAEGEAVVVSEPMLLDTATPDLLSVTVEAGGRLVFD
PAADLAKLAAEWIKVAGELHVGAEDCPFTGQAEIELLGKKNVTVGVQVHEHGAEFKA
LVVVDGGRLEVHGQPRQAWTRLQTTLPQTTRELVVGDALFNHRETENVAHWYRGF
MGYQFNSTGQPVRYMRLRRSCDFSDLVTMQGSVVLLVVQRKVELDRDQQLEHAADA
FEQVCYGGGQRSHLRDLSLGETVAWAAICHVGHPENTIEELGHPEGDNSVTSRLVKQV
GDVEYLAQSVTRLTSDWPYEIDMLTYTAGNVPDAHIVLDVSSNVSDWRAGDTVAITS
TDYDWRQAEEFTLLDCPDCSATQIMIGWPVRWTHWGETTDGVNMGAEVGLLNRNV
RIHGRMEDACYGDNHCGRFNFDTFGGQIKVFANFASVRIVNAEIFHMGQNSVLGAYPI
HFHMCGDVDTTRELRAYVRSNAIHHTFARCVTVHGTSGLLVEGNMAYDHFGHCFFLE
DGSEKRTVFRNNLGFSTRRTTLTPSDHGRASTFWITNPLTTMEGNVAGGSEGWGIWY
TFPRKPIGPSAALDVMEHNEAMKTPLDPFIDNVAHSNNEYGLHFDGVLRSNGHIHGG
FNYDPREDPLNATSPTVVVTLDGFTAFKNYKSVWLRMSRVVAKRFNIAESVEGVVFA
WSGAGEKRLEDSLIVGETANKGRPEGWIRLTNGTSIWWDRQIRMNGADNALPIIGVIF
HDTIMSLNRVTFDRFESNIVRPSGGIGMRRKIRHYTSPVNSVSGLVFRYADPLEGNRAF
DASEGRGFADLAGNIQNVIRDLDGSLTTYANSTVVKPYDIHLNARCAPVPQWNLYVCP
TMFTKLLFMWWDRSVGNLNTFLTRNDGSATFTYGVNFQNSYALNSQEGYIVHFNTS
VPRYFKIRLAGVEAGVVQTVGLCIGKGVTPLTVEMNSVPVPSFDSLDDQGADVHWYL
DTEAGVLFFRFSSPYHRTADTVDECPGQDGKPAGCPEVLVVLPSSNTDGDCSGRAYPA
YS

>CL11.Contig4_Ba_mix
MNSAGPNYPMASLYVGDLHPDVTEAMLFEKFSTAGPVLSIRVCRDMITRRSLGYAYV

NFQQSADAERALDTMNFDVVKGRPIRIMWSQRDPSLRRSGVGNIFIKSLDKSIDNKAM
YDTFSAFGNIMSCRVATDENGVSKGYGFVHFETEKAANDAIEKVNGMLLNDKKVFVG
KFVPRKEREKELGEKAKRFTNVYVKNFGEDLDDTKLLEMFEPFGTISSHKVASGEDGK
AKGFGFVSFEDPEAAEKAVNEMNGKEINGKTIYVGRAQKKAERQHELKRKFEQIKLER
MNRYQGVNLYVKNLDDGIDDERLRREFAQFGTITSARVMMEDGRSRGFGFVCFSSPE
EATKAVTEMNGRIVSSKPLYVALAQRKEDRKAHLASQYMQRMTSMRMPGVNPMFQ
AGAGGYFVPTMPQAAQRFYPPAQMAHQMRPGPRWQQPAGGQMRLGGQAAGGMPA
PFRGQAQRAAGGQAPAGGMRGMQAGQTRPMAQQALLPAGMQPRPMAPQPGAAGQ
QPRPAYKYTANMRNLQQLSAAGVSGQAPLMPGAAQALAMQQQPAVRVQGQEPLTAS
MLAAAPEQEQKQMLGERLYPMIQRMYPELAGKVTGMLLEIDNSELVHMLEHHESLK
GKVDEAVAVLQAHQAKATPA

>CL14024.Contig1_Ba_mix
MVQRLTYRRRLSYNTRSNRVKVVKTPGGRLVYHYRKKPRRVPRCADTGKKLTGIRSA
RPQEKKRMKRKHKTVNRTYGGVLCHSAVRERIIRAFLVEEQKIVRKVLKAQQASAKA
K

>CL15766.Contig1_Ba_mix
MLMPKKDRVKIYEYLFKEGVMVAQKNPHRPRHPNVPVPNLYVMKALQSLKSRGYVS
EQFAWRHYYWTLTNEGITYLRDFLHLPSEIVPATLKRPTRAETARPRAAAPRPDGGK
MEGDRAAYRRVGEGGPDKKGEAGAGAGQMEFRGGFGRGRGMPQ

>Unigene36236_Ba_mix
MLMPKKDRVKIYEYLFKEGVMVAQKNPHRPRHPNVPVPNLYVMKALQSLKSRGYVS
EQFAWRHYYWTLTNEGITYLRDFLHLPSEIVPATLKRPTRAETARPRAAAPRPDGAK
TEGDRAAYRRAGEGGPDKKGEAGAGAGQMEFRGGFGRGRGMPQ

>CL4543.Contig1_Ba_mix
MVVLGEVFPNFEAESTEGHIRFHDFIRGSWCILLAHPSDFTPVCTTEMGAAAKMAPAL
LRRGVKMACISCDPVSSHKTWIEDIRAYNGLGSGPFPFPIISDEERQLAKKLGMIDPDE
PRFDNKPATARAVFIIGPDLRLKLSMLYPGTTGRNFAEIQRIMESLLVTAKHKVVTPEQ
WQVGEKCFVVPFLSMEEAKDMFPAGVQVHPVPSGKQYLRTTPMP

>CL2680.Contig1_Ba_mix
KCFYLPQVLURSLSLLGRMSALSVLRCRLAAGRPPALCSPRLLPPAGRTVWRGPAAHS
RALVVLDDLRQLPQSPRPALLLGAAGLLPFAAAPVLMLRTGHFSAELLHAQLAYGACI
LSFLGGVRWGVTLPDGAPQRPDWANLTRSVTPSLLAWAGLLAPPSAGGLMLIGGLGL
AGYWDMVMYGYPAWFKALRFCLSVGAILSLWTAL

>CL14396.Contig1_Ba_mix
EGLLALLDESTLSEKASDQYFVENCKETIESRHFFPEKDGFAIAHYAGKVSYSNKDIVA
KNKDYLDPEVFDTLRQSENPFIAPMFLLPLTKTGNLTMDREQAQREAGGKARPVKLN
DDVGNKRFDTRSRGRLSQTSLVLLTQASCYRYSAMEVMHKLINSTAHFVRCVRSNSEG
AADTWDTGLVAHQLKCMQIANTIAIRKDGYSQRIDFAEFLRRYQFLAFDFDETVEVTR

ENCRLLMVRLKLDGYRMGRQKIFLKYFNEEYMSRLYEHEVKKIAKVQAMIRAFLVRR
R

>CL9899.Contig1_Ba_mix
EGLLALLDESTLSEKASDQYFVENCKETIESRHFFPEKDGFAIAHYAGKVSYSNKDIVA
KNKDYLDPEVFDTLRQSENPFIAPMFLLPLTKTGNLTMDREQAQREAGGKARPVKLN
DDVGNKRFDTRSRGRLSQTSLVLLTQASCYRYSAMEVMHKLINSTAHFVRCVRSNSEG
AADTWDTGLVAHQLKCMQIANTIAIRKDGYSQRIDFAEFLRRYQFLAFDFDETVEVTR
ENCRLLMVRLKLDGYRMGRQKIFLKYFNEEYMSRLYEHEVKKIAKVQAMIRAFLVRR
R

>Unigene5067_Ba_mix
EGLLALLDESTLSEKASDQYFVENCKETIESRHFFPEKDGFAIAHYAGKVSYSNKDIVA
KNKDYLDPEVFDTLRQSENPFIAPMFLLPLTKTGNLTMDREQAQREAGGKARPVKLN
DDVGNKRFDTRSRGRLSQTSLVLLTQASCYRYSAMEVMHKLINSTAHFVRCVRSNSEG
AADTWDTGLVAHQLKCMQIANTIAIRKDGYSQRIDFAEFLRRYQFLAFDFDETVEVTR
ENCRLLMVRLKLDGYRMGRQKIFLKYFNEEYMSRLYEHEVKKIAKVQAMIRAFLVRR
R

>CL888.Contig1_Ba_mix
ALGVPALGQAGFECPDQLPGFFPHDVSCDKYWACEEGVATLKTCGNGLAFDDTDPNY
EKENCDYLHNVQCGQRTELEPPISSPNCPRQYGIFADEANCNTFWSCWAGEANKYEC
PPGLAYDSEQRVCVWADQVAACKKSEKEGDFQCPAEGNGLGTYSLYAHQEDCRLYYV
CMGGAPREYGCPLGTVFKIGDLDGSGQCADPADVPGCEEYYGDVDIRALRALGF

>CL6240.Contig1_Ba_mix
MYRIPGLLRGPAARQLTRCFAKDVRFGGDVRASMLQGVDVLADAVAVTMGPKGRNVI
LEQSWGSPKITKDGVTVAKAIELKDKFQNIGAKLVQDVANNTNDEAGDGTTTATVLA
RAIAKEGFEKISKGANPTEVRKGVMLAVQAVVANLKNMSKAVTTPEEIAQVASISANG
DKTIGDLISSAMKKVGRDGVITVKDGKTLHDELEVIEGMKFDRGYISPYFINSSKGAKV
EYQDALVLLCEKKISNIQQIIPTLELANTARKPLVIIAEDVDGEALTTLVVNRLKIGLQV
VAVKAPGFGDNRKNTLQDMAIATGSIVFGDEASTIKLEDVQLSDLGQIGEVQITKDDTL
MLKGRGNKTDIEQRCVQLKDQIEETNSEYEKEKLQERLARLASGVGVLKVGGSSEVEV
NEKKDRVTDALNATRAAVEEGIVPGGGVALLRSATALSALQPANEDQKTGVEIVRRAL
RMPLQTIAANAGVDAAVVVARVQDAQPEIGYDALNDEMVNMMERGIIDPTKVVRTA
LTDAAGVASLLTTAEAVVCELPKEDKADAMGGGMGGMGGMGGMGGMGGM

>Unigene11234_Ba_mix
MASLNPVHVLRGGAEEEKAEIARLSSFVGAIAIGDLVKSTLGPKGMDKILISVGRSEGKV
EVTNDGATILKNIGVDNPAAKVLVNMSCVQDDEVGDGTTSVTVLASELLKEAEKLVG
MKLHPQNIIAGWRRATDIARKALAGSTVNHGSDPDKFRNDLMNIARTTLGSKILAQH
KDFFAELAVNAVLRLKSSGNLQAIQVLKVQGKTLRDSFLDEGFILNKKIGVHQPKRME
NAQILIANTPMDTDKIKVFGSRVKADSTARVAELEVAEKEKMKDKVEKIVKHGINVFI
NRQLIYNYPEQLFADAGVMAIEHADFDGIERLALVTGGEIVSTFDHPELVKLGSCKVIE

EVFIGEEKMIKFSGVPLGEACTVVLRGATQQILDEAERSLHDALCVLTSTIKETRTVFGG
GCSETLMAVAVLEEAARTPGKEAVAMEAYARALLQLPTIIADNGGYDSALLVSQLRAG
HTQGQNRLGINMLDGTVGNMEELGITESFMVKQQMVASASEAAEMILRVDDIIKAAP
RKRVDDRGHC

>CL2410.Contig1_Ba_mix
EREDSVYKAKLAEQAERYEEMVEAMQTLACLDVELTVEERNLLSVAYKNVIGARRAS
WRIISSIEQKEETKSAEEKLEMIRSYRIKVEKELKDKCGEILQLLDSHVIPSATTEESKVF
FHKMKGDYHRYLAEFATGNERKDAAENSLLAYKAATDIAMTELPPTHPIRLGLALNF
SVFYYEILNSPDRACRLAKAAFDDAITELDRLSEENYKDSTLIMQLLRDNLTLWTSDM
QTDGKEGEAGTGEEAPAVQDVEEQDVA

>CL8957.Contig1_Ba_mix
AIPFRMLASSASRLLRGLSASVRPVRLLSRTAPALAPRVQQPAPDFKGQAVVDGEFRT
VQLSDFKGQYLVLFFYPLDFTFVCPTELTAFSDRMDEFRALNTAVVGVSTDSQFSHLA
WIDTPRRSGGLGGLSYPLLSDFSKAIAREYGVLLEDEGVALRGLFLIDPSGVLRQMSVN
DLPVGRSVDETLRLVRAFQFVEEHGEVCPANWQPGGATIKPSPAESKEYFEKV

>CL2356.Contig1_Ba_mix
MGIDIYAIKPSPPCRAAFLVAKAIDLDYNLKIVSLEKGENRTPEFLKMNPAHTVPVMTD
GELSIGDSKAIITYMMNQYAPANLQSLYPRDPAARALVDQRLYFDSQLFASLRGIVFPL
VFMKDLKQSQANMPRVDENMALLETFLTRSTYLAGEHLTIADLATLANVSSVEAGGL
DMSRWPHVQAWLAKLKAELPYYEQANGEGAQMMGAMYKNALKE

>CL4955.Contig1_Ba_mix
MGIDIYAIKPSPPCRAVFLVAKAIGLDYNLKIVDMEKGENRTPEFLKMNPAHTVPVMT
DGELSIGDSKAIITYMMNQYAPANLQSLYPRDPAARALVDQRLYFDSQLFASLRGIVFP
LVFMKDLKQSQANMPRVDENMALLETFLTRSTYLAGEHLTIADLAMLSNVSSVETGG
LDMSRWPHVQAWLAKLKAELPYYEQANGEGAQMMGAMYKNALKE

>CL4041.Contig1_Ba_mix
MCGIFAYLNHLTPKDRKEVFQILIQGLQRLEYRGYDSAGVGVDGKDGSIEIYRAKGKVK
NLEDLIMSEDNKQDMSEILKTHVGISHTRWATHGVPAPVNAHPQPSDASKQFICVHN
GIITNYKDIKQFLIAKNHVFESETDTEIISKLTLHMYRDHPNHTFQQIVELVIQQLEGAF
ALCFKSVHFPNQVVAARRGSPLLVAIKTKEHLATDHIPIMYSKEDFKGENTKSRKISPA
DARHVPAGTHGGNVPLIPRTESTAEFLPTGDSQEIEYFLASDASAVIEHTNRVIYLEDD
DVAWIRDGMLSIHRLKRESMDECSTREVHTLKMEIQEIMKGSFSSFMQKEIFEQPESV
VNTMRGRINFEDETVVLGGIKDYLPEIKRCRRLILIACGTSFHSAIATRQLLEELTELPV
MIDLASDFLDRKTPIFRDDVCFFISQSGETADTLMALRYCKSRGALIVGITNTVGSSICR
ESHCGVHINAGPEIGVASTKAYTSQFISLVMFALVVSEDRLSMLPRRREIIQGLKVLPEL
IKEVLALDSKIEELATGLKGARSMLVMGRGYNYATCLEGALKVKEITYMHSEGILAGEL
KHGPLALVDPGMPIVLICTRDPTYVKCMNALQQVTAREGRPILICSKGDTEPARFSNAL
LEIPHAVDCLQGLLTVIPLQLLSYHLAVLRGCNVDCPRNLAKSVTVE

>CL14928.Contig1_Ba_mix
LLVACLGAIQAQKMHITLKTKDAPEQNPNLTPKEDAPSFHVQLLKNIKAKGVQTFVGE
ISLGTPPQLMSVSVETSSALSYFPSVRCEDQFCKTRRQYNSSASSTYEALGTPFELPPGI
NASGVLSKDSISFAGITVEGVVFGEAESMWNKWLSILPPDGVLGLGLQGMAPEGVRPIL
DVMAEQGVISERVFGMWLGRDPQGGELTLGGLNDQLYRGELTWANLTPPDGQWWT
APADSIVLDGLTQLDLCPGGHCVVLPASNSPYFIVSEAQAAAINEALGGLDIGQPGAAGL
DCTTLYRLPNLTITVGGRAMVLGPLEYTFLLRFEGGLQMCVSGFLGFPGVDDHTMLLG
TLFMQKYYIAFDRENNRLGFADSA

>CL16649.Contig1_Ba_mix
LLAACLGAIQAQKMHITLKTKDAPEQNPNLTPKEDAPSFHVQLLKNIKAKGVQTFVGE
ISLGTPPQLMSVSVETSSALSYFPSVRCEDQFCKSRRQYNSSASSTYEALGTPFELPPGI
NASGVLSKDSISFAGITVEGVVFGEAESMWNKWLSILPPDGVLGLGLQGMAPEGVRPIL
DVMAEQGVISERVFGMWLGRDPQGGELTLGGLNDQLYRGELTWANLTPPDGQWWT
APADSIVLDGLTQLDLCPGGRCVVLPASNSPYFIVSEAQAAAINEALGGLDIGQPGAAGL
DCTTLYRLPNLTITVGGRAMVLGPLEYTFLLRFEGGLQMCVSGFLGFPGVDDHTMLLG
TLFMQKYYIAFDRENNRLGFADSA

>CL1199.Contig2_Ba_mix
ISGVDCLLFAIGRDPNVELDLDKTGVTLEKGHIKVDAFQNTSAPGVYALGDVCGRALLT
PVAIAAGRRLAHRLFDGKTDLKLDYNNIPSVVFSHPPSGSVGLTEREARDKFGDAAVR
VYRSSFAPLYYALSERKVKTHMKLVCAGDEQRVVGLHMVGQAVDEMLQGFAVAVKM
GATKAQFDDCVAIHPTSAEELVTMR

>CL3558.Contig1_Ba_mix
EVPPQLEDLLLEFTVNVLVNQPDNLVAYAVEYFTRLNESGNKYSGANSKLAPADTNN
VQDGGVSDQDDSMMSEDEGPPQNCNYRRKSVFAEAYNPEEDDDEGQKAVFPKSDEQ
RARLSDAVQGILLFRALDPHQMQEVIDAMFERKVTAGENVIQQGDDGDNFYVIESGRY
SIYVKMDDSAEPKKVGSYDNKGSFGELALMYNMPRAATITADEEGSLWAMDRQTFR
RIVLKNAFKKRKMYESLLETVPMLKALEPYERMNLADALVPRTFTDGELIIEQGDEA

>CL3.Contig8_Ba_mix
GSGRVCRPRDDSCRPAMSAGNCPSGSYCGQQGRCTPLLDVNAPCWNDVQCLHHVCR
GHVCKMDACPQLDSDQDCPEGQFCSSSQRGNICIDRRPDGEACSSDAQCQSYRCDGRR
CSRDTCKTLLSRDGCPDGHICQQGQHGNLCVPLLARGASCSSDRSCLSDSCRAGRC

>CL3.Contig20_Ba_mix
GSGRVCRPRDDSCRPAMSAGNCPSGSYCGQQGRCTPLLDVNAPCWNDVQCLHHVCR
GHVCKMDACPQLDSDQDCPEGQFCSSSQRGNICIDRRPDGEACSSDAQCQSYRCDGRR
CSRDTCKTLLSRDGCPDGHICQQGQHGNLCVPLLARGASCSSDRSCLSDSCRAGRC

>CL3.Contig33_Ba_mix
GSGRVCRPRDDSCRPAMSAGNCPSGSYCGQQGRCTPLLDVNAPCWNDVQCLHHVCR
GHVCKMDACPQLDSDQDCPEGQFCSSSQRGNICIDRRPDGEACSSDAQCQSYRCDGRR

CSRDTCKTLLSRDGCPDGHICQQGQHGNLCVPLLARGASCSSDRSCLSDSCRAGRC

>CL3.Contig21_Ba_mix
CGKSQICVHTLIGNECMTRLPLGAQCTSNLQCLTNICRDNHCRRPLAQNGDRCRADND
CLSRNCRRGTCRPPPAQDGDRCRSNKDCLSRNCFKGTCSPPGTGGGGSRCQSNEDCLL
GFVCSQGICSQLGGDGDFCRGNRECQLDLVCLDGICGPPRTADNDGRFCQFNNDCLLG
LVCISGICSETGGEGGICQLNSDCLLGFSCLDGLCVGDGDGGGDGGDGGTVDVDTLLGIG
EQCFAAIECFSGRCAGGQCAEPLLGIGDDCSTAESMPSNALCR

>CL336.Contig1_Ba_mix
GVTDNQYRYIDAEGTVRTVKYWADTPSLRSSGAHTPHSPQLRMAAPAPAPVQDTPEV
AAAKAQFMATFRMAQQGVIPAPVQDTPEVAAAKAEFMAKFRAAQRAQRKRRSVTVG
LSHTPASTALLP

>CL336.Contig5_Ba_mix
GVTDNQYRYIDAEGTVRTVKYWADTPSLRSSGAHTPHSPQLRMAAPAPAPVQDTPEV
AAAKAQFMATFRMAQQGVIPAPVQDTPEVAAAKAEFMAKFRAAQRAQRKRRSVTVG
LSHTPASTALLPGAYWKWSPLY

>CL1239.Contig2_Ba_mix
GAMIPVLLLVSLVGHVAGHGRLMDPPARNAMWRFNFSNPVNYNDNELFCGGFVVQY
QQNDGKCGVCGDNYALDEPRPHEAGGEFAKGLIGKRYSPGQLIDIEVELTANHMGNFV
IRLCPHNNDRKTVTQECLNKHVLRVAGTNSIRYVIPEDSGKAGIFRWKVELPPYLTCT
QCVMQWTYYAGNTWGTCSDGEQAVGCGPQETFINCADIAIVSNTPYFGPVHNSLDRT
LPLTKSEVARVVQEDLDNEPRAPAFRPQICLPHGEFKQEEGADRFCLRCMVNPEACPT
DRCMCVTECEAQGEFAKRPQADLFCHENCLGFPSHCPEDKCRCF

>Unigene4984_Ba_mix
LVRHAAGHGRLMDPPARNALWRFGFKGPVNYNDNELYCGGFVVQYEQNGGKCGVCG
DNYASETPRPHEAGGEFANGVITRRYAPGQIIDVEAELTANHKGNFVIRLCPLNNPNE
TVTQECLNKHVLRLAGSKAIRFVIPEDSKKSETFRWKVELPPYLTCTQCVMQWTYYA
GNTWGTCSDGEQAVGCGPQETFINCADIAIVSNTPYFGPVHNSLDRTLPLTKSEVARV
VQEDLDNEPRAPAFRPQICLPHGEFKQEEGADRFCLRCMVNPEACPTDRCMCVTECE
AQGEFAKRPQADLFCHENCLGFPSHCPEDKCRCF

>CL888.Contig2_Ba_mix
ALGVPALGQAGFECPDQLPGFFPHDVSCDKYWACEEGVATLKTCGNGLAFDDTDPNY
EKENCDYLHNVQCGQRTELEPPISSPNCPRQYGIFADEANCNTFWSCWAGEANKYEC
PPGLAYDSEQRVCVWADQVAACKKSEKEGDFQCPAEGNGLGTYSLYAHQEDCRLYYV
CMGGAPREYGCPLGTVFKIGDLDGSGQCADPADVPGCEEYYGDLDVKELAMRALGGG
SGTSG

>CL21.Contig3_Ba_mix
RRFLCVVALAVVVADASSVRERRQALRKKDSAKLEELCRDRGDEYFRLEDENDCKVV

YRCTRGGLKRIQCPDGLAFDIRQQTCLWKRHVKNCDLLSKERKVLPLLKTDEPLCPVD
QLACGSGDCIDSFLFCDGQPDCADKSDENACEVGVDPNGSFLCDTSQCLLPSCFCSSDG
TQIPENLEPAQTPQMITMTFTGAVNVDNINLYQEIFRDERQNPNGCTAKGTFFVSHKY
TNYSAVQELHRKGHEIGVFSITTNDDPSYWSDGSYEIWRDEMAGARDIIENFANITDG
TVAGVRAPQLRVGGNNQFAMMIDQQFVYDSSITAPYGRVPIWPYTLDYQMPHKCLGT
SQNCPSQSHPVWEMVINELDLRENPETEERLPGCHLLSSCASINNPEQFAKILRNNFE
HHYSTNRAPFGLHLTADWLQNNKGFINELIKFIDEMQAKNDVYFVTESQVISWMQAP
KETNAIRDFDDWKLKCEVKGLPECSIPNACLLTTRELPGDNFYLNTCMECPKNYPWI
KDYTGNGFA

>CL14958.Contig1_Ba_mix
MQLAAALVVLLAVSANGLRVKRQDAEATEDEAVQAICLGKGPGEWFRLNAGSASCNV
VVQCTSAGLQQIKCPHGLAFDLNKQACDWKDTVSDCDQTQRPKLVLPRINTDEPLCA
TQDELACGDSTCIARSLFCDGKPDCADSSDENICDAESDPNRAPPCDPAQCQLPSCFCS
ETGNQIPGGLAPEQVPQMIMITFNGAINNNNIDLFDDLFSPSRLNPNGCPMKGTFFVS
HKYTNYTAVQDLHNRGHEIGVFSITQNDTANYWTNASTEEWAREMAGQRYIIEQFAG
IADNSVVGMRTPYLRVGGNNQFSMMDDQTFLYDSTITAPLGDLPIWPYTLLNKMPHP
CHGNAQKCPTRAFPVWEMVMNELDRREDPRIDDNLPGCAQVDSCSNILNGEQFYNF
LNHNFDRHYLTNRAPLSLNFHSAWLKKGDFLDYLLRWIEDIQISVTDVYFVTMTQVIE
WMQEPLDVQQTKTFASWLTKCDESTGFECTKPGGSNCALHTDGVAGTFNMQTCKRC
PPNFPWLNDPSGKGYF

>CL8680.Contig1_Ba_mix
LDRIPGCTLPEQPACQPREKFRRMNGECNSDAEPRLGRAQTAFSRFFPAEYEDGIWQP
RSKALSGDPLPSARLVSERVVRTTPVMDPWSSLSVMQLGQILNHDIISTAAFTLENGG
GLFCCNRDNTMPAEPLHPLGCNPIPVPADDPWYAQFGRTCMNQVRSLPAPFENCIAG
PTGQLNQVTHFLDLSTVYGSSDTDMRRLRLNAGGLFKMSEGNLLPQSGGRFISGEGRL
SENPGLAFMHTVWTREHNRVANTLSRLHRHWSDEQLFQEARRIVIGEYQHIIYNEYL
PVILGFKYTREHGLDPEFGHSFSYTNVIDPRVTAEFSTAAFRFGHAQIPDVFQLMTQNG
MNLTAIAMEDSFFREDMLKLPNFMTNLATTLTRQKQRMVDNTFSPAVHEKLFASGQ
PSGLDLIALNINRGRDHGLPGYATVLENCLERTLSGGWDEMSEFFEPDVLRQLRAVYQ
SVFDIDLYIGGVSEKKVPGALVGPAFQCIIGEQFFATRYGDRYFYDNGDMPHSLTFTQV
REIQKASWARILCDNVEDVNVMQPLAFFAPNPLFNAVRHCRSFAIPTVDLRAF

>Unigene25886_Ba_mix
GAVKGLKDKLLLSGSVRDYHFVSQGKTTIPGVDDKEEMEATDEAFDILGFTQQEKENI
YRIVAAVMHLGTMKFKQRGREEQAEADGAEEGERVAKL

>Unigene15759_Ba_mix
VSQHHPAGSTVEAYLAAMQTQWSWILQLTLCLETHLKHAQLSHKLFKDVEDMTRVI
NQQDDRLNSEFSRSEFTLDDGEQLLRDMQLLRDAMAQQAEEIERLERRAQQATPLRQ
RRHPDTGVRLTTICNYK

>CL7850.Contig1_Ba_mix

ISDQEKVRIVSDFILHSPPGEFNEVFNDVRHLINNDNLLKEGASGAFVQYNKDQLTPVR
LPGSDYPAFITDFNDLGGSRFFDPRTKQSFRFDHLKKEASDLKPHEGDSTAEPWRRAL
EAEMTAYTADHYKNGACAVFGHSNDGDVTLVACIEDHQFQPKNFWNGRWRSQWTV
TIPSSGTAQLKGVLKVQVHYYEDGNVQLVSSKDAADGAVVASESQTAKEVVRLIETSE
NDYQTAICENYQTMSETTFKALRRQLPVTRCKIDWGKIIQYGIGRELKQQ

>CL2450.Contig1_Ba_mix
PPTEAPPTTAAPTTAAPEGGSRSSPVAGGPPPLGGSRSSPVAGGPPPPGGSRSTPMAGG
PPPPMPGSRSTPMSGGPPALPESPKTPTVGIADEPDFVIPDDIDLPDLDLPPYDPNFFF
DYPPEPLPDTYLIDLILPPEQPDWPPP

>CL2677.Contig1_Ba_mix
REQGGKHQDGVYWFTGMPVPVYCDFSHDGGGWTLLLTAVSADGWDALSALGRSRRS
PTLTDNYSILAHADAIRDLGHSARFAYRIEAQVETGRQRWGGVWLAPRHYSFIHESPS
QTDVTIVRKFDEWEYKDIGIEKRMPWLSTDSAHDAVLTTSASVSVEWWGTLVSHKT
HTYYKHSPWLNGE

>CL2236.Contig1_Ba_mix
LKLLQIALCLTCLGLIRNYGLAFGGSSTGDALNRQLVGIIACGGMVLVTAPLLIALVAGEI
AIEKSFLETLYNVVGFIMMITAGIMAVEFYSNFGSSETRDAGLAMGSLAIINAIIYLVDAF
VAFR

>CL7397.Contig1_Ba_mix
LRLLSTSPSCGSESDGWLSKLLVRKIEPTKESHSRMLSDKEVIYELQTHNVRCGEMDK
VPSTTTAATTARWPSGRPPASWPRSWSAAGRSPWATRTSASTCGSTTADTRPVDDA
RNAHIDDKSLAALVKERGTLLRARHSQYLLAFSFWPAPAPRSGKHLYEIRSYLLKPGT
MIEWGNNWARAIHFRREGDEAFAGFFSQVGRLYNVHHLWCYDSMAHRKEIRETAW
RKPGWDECVAYTVPLITNFQSRIIVPTPFSPTR

>Unigene5246_Ba_mix
RLVSSLGLRLLSTSPSCGSESDGWLSKLLVRKIEPTKESHSRMLSDKEVIYELQTHNVR
CGEMDKYIDNYGRYHGEVAERTAAGQLAQELVGSWEVTVGDQDQCIHLWKYNGGYK
AVDDARNAHIDDKSLAALVKERGTLLRARHSQYLLAFSFWPAPAPRSGKHLYEIRSYL
LKPGTMIEWGNNWARAIHFRREGDEAFAGFFSQVGRLYNVHHLWCYNDLQHRKET
RESAWLRPGWDECVAHTVPLIGGMQSRIMWANPFSPTQ

>CL9979.Contig1_Ba_mix
DSKFDIGLVLASFKKCGEDTGGSLTMDDYIAGYVELNKFFGLLGTVFGFVGSDVHQKLV
QLECLRKGGASDSYVTVERMILYEKQNGVFESDKAGNGCRTLLRLHRALAFIADFLRE
LHDRSDEQKMGSVCQASYKRTLAVHHPWLVQQGALLAMHMLGTRGDIVEKAVGGDS
SDKEKHKDTLLECIDVMQAVYADTQKLYEQH

>CL7745.Contig1_Ba_mix
VVTGCSRGIGLAMVSELVKNNFKVFATCRNPASASQLSGLLTSLEQPAAVALDVTDEA

SIQAAYGTVKEAAGRVDLLINNAGISVKTHPVDPCETAQLSELNRLLETNVGGLVRVTQ
VFLPLLRASPRPRVVNISSLLGSIGVCKERGALAPDHFFSYRISKAAGNMATVAMSTAIT
EVEFLAVHPGWVATDMGNSGGRTADITVEESAEGVVREALRPQRRTGEFVDWRGNP
VPW

>CL2357.Contig1_Ba_mix
LKYAVGLAPSDPDSQPVMILGQIRHLARVSWDTIKVKLEPQVSEAVWQRAVSELFPSP
TDSLHLYLNQATLAALPGKCSRHNTPSRAHSVTRLVRSLLTGADQTVVLVCERADML
ATGCAVARALPLFTRKSGPQPEPTITVEVLVVDGEQASAEEVQLMQDAARGIRLTARL
VDTPANEMHTDAFVEEVKKVGEELGVSLTLIRGEDLKARGFGGIYGVGKAAVHPPALA
ILSHTPAGATQSIAWVGKGIVYDTGGLSMKTKTTMPGMKRDLGGAAAILGAFLVTVKA
GFTQNLHAVFCLAENAVGPIATKPDDIHTLYSGRTVEINNTDAEGRLVLADGVAYANK
DLKCTTILDMATLTGAQGTATGKYHGALLTNEDTWEVLGAAAGRASGDLLFPVPYTP
ELHFSEFASSVADMKNSAADRSNALVSCAGLFIAANLGFDFPGSWIHV

>CL2646.Contig1_Ba_mix
FSRPSPYLDGARTQHVSDFKYTRYQNQINGSVPTGAAGATDSVKVERRTKIVDKKTIGI
ALYGLGRAGVIHLGNLMINPRVSLVYTLESDQARYDAMKERWGDRMPPNVLPADSGT
VLADPRVDAVMVATPTHTHFDIIQQALLADKLVFSEKPIGENEQQARRCYQLAEQRGL
SVFCAFNRRFDPALQTVRSGVRRGDVGVVHTIKTVARDSPLPSIDFLRQSGGIFHDCAV
HDIDLICWILGQFPLRVLASASAHVPEIAALGDHDTAAIVMEFEGGTIGSIDLSRNAVYG
YDQRTEVFGPGGLLTAGNERPVNVHHLTSGGETAVPFYFSFPSRYSESYANELDSFLDY
VQGKIPLPIRPEDSLRVSQIASACEESLRTRAFVELDY

>Unigene34863_Ba_mix
RSQKVEILLDIQSVRSPDAWVPCNGAIYLSICLLGQYIRTKDKAPIFPINFNETFVVEKVF
GHVFRLADLDDMLDAEVVYLELIQRPPAGPDTVLASFETSARELLFP

>CL74.Contig3_Ba_mix
DADKQSGDVTCMRFHNGKLYTGAEGGKLIVYNPDLTIYKTMQAQFNTINAICFFQGDL
VTTGNDGLIKVWDSSSLELKKTLEGHENEVRVLKARDDKLFSGDMNGKVKIWDKNY
TMLFTLSVVEEVWSMDMARDIIYTARCNDVTCTQIGFKEMSVPGETKFIILDTFEGRG
PMCIAGEHIIYTNREGTVLLVKENKPGHKHVNNMTGHEKIINALIYTDNLVYSAGYDG
MVKIWDGTTYKPVASGTTVGNPVFSLGVGDNGEVYAGLNDGSI

>CL74.Contig5_Ba_mix
DADKQSGDVTCMRFHNGKLYTGAEGGKLIVYNPDLTIYKTMQAQFNTINAICFFQGDL
VTTGNDGLIKVWDSSSLELKKTLEGHENEVRVLKARDDKLFSGDMNGKVKIWDKNY
TMLFTLSVVEEVWSMDMARDIIYTARCNDVTCTQIGFKEMSVPGETKFIILDTFEGRG
PMCIAGEHIIYTNREGTVLLVKENKPGHKHVNNMTGHEKIINALIYTDNLVYSAGYDG
MVKIWDGTTYKPVASGTTVGNPVFSLGVGDNGEVYAGLNDGSI

>CL74.Contig6_Ba_mix
DADKQSGDVTCMRFHNGKLYTGAEGGKLIVYNPDLTIYKTMQAQFNTINAICFFQGDL

VTTGNDGLIKVWDSSSLELKKTLEGHENEVRVLKARDDKLFSGDMNGKVKIWDKNY
TMLFTLSVVEEVWSMDMARDIIYTARCNDVTCTQIGFKEMSVPGETKFIILDTFEGRG
PMCIAGEHIIYTNREGTVLLVKENKPGHKHVNNMTGHEKIINALIYTDNLVYSAGYDG
MVKIWDGTTYKPVASGTTVGNPVFSLGVGDNGEVYAGLNDGSI

>CL14828.Contig1_Ba_mix
MPVTLDTYLPSEQELTVEELNIGYPALKAGSFHLGKYCEAQRDEFMLCRQEEKDPRKC
LAEGRAVTSCALEFFRKVKSTCLDEFNQYANCLDRGSPDLKFRMCRNTQSVFDKCVQ
DNMGMERPYYGYFCAPKVIRTNRPRPAPEPPLEFPNTPDELPDTMPRKPAPY

>Unigene9033_Ba_mix
XGLVQQISNQAMYQYGAEAACYSGQRLYEESCGELLRSPGFENWSYENRIIDGSVVPQ
HRDIQTD

>CL46.Contig1_Ba_mix
TQKNALEVHSPHCKYKADESRPNLILLSDPQRKKRSAPEAPAEDQQADAAMFYRSYR
YPAFGYTYPGVRILKKRAADPEKEEEDEKKKVVYINAMPYSSLGYPFYSYAPYIYKK

>Unigene11352_Ba_mix
XNHNSSQLTMKCIVLLFAVVSCASAYYFVTPSAYYGGVSPYIWRKKRSAPEAPAEDQQ
ADAAMFYRSYRYPAFGYTYPGVRILKKRAADPEKEEEDEKKKVVYINAMPYSSLGYPF
YSYAPYIYKK

>CL1824.Contig3_Ba_mix
XSMPQPASTGGLSYAVQNLYGGYYYVIVNNEAVPVGFSATQPSGDWKSLGQMSVSACL
GTCYHGDGLFMMVQTVDGVTSCWSSSSVDMGQLSQTVPSAPGAYEVFDISSMRIGSQ
MAVSHSSGFVVKSTAAKNGYYTFDVTMPDKEESSELTIYYTQSAPSSLQLFPMSEGHP
AECVASAMGMMCDVAIWNPSDPEKPCQCGQLINGGPLGLTDTSVPDVAGYMEVIQLR
AMQPPADFEGLSYTMYDLFAGYFRLIVNYKALAVRFSGSEPAGNWKNLGHLSVSECL
DACYQGDGLFMMVQTMNDVTSCWSASLVDMSLLSREYPSGPGEYEILDISPMKIGSEN
TVTHSNGLVVTSTGIQNGYRAFTVTMPGTSFPSELTVYYTQSAPSSLRLLPVSSAQPAK
CVSSATLMLCDVAVWDPNNSDMPCQCGEIVEGETLGLTDTSVPDTVGFVEVIQLIPMP
PPDVEPGHASFQSFWMTPEVYRLILNGEIIPASFARSAAVQAAAGVNLDRAIVKEACAT
TCFGLHYNGAALSHQTCRCLEDPSSVSSQQQYVAGDDLVSLINFAKYQPKPDGSSSTIT
LQVPGTRYGRFLFGNAVLPCGFTPQNVDPSTGPPGSWKDAGERTGLRDCASECSKLQL
NFGAMLPLGKNIFKCWCAESVDMSAFSTAMPEVGGRAQILDFTDVIRGISSSRLQIRHV

>CL2298.Contig1_Ba_mix
XLQSQAWRRLPAVPPLFCLQMCALRGYTYAAVGNSEDPTCDCSKRSPNEADLFRTAK
TDSDRQVFFLKGLPRIGKMSLVYVTENSFLQFIFSGTSAARPIVLYLRSAPPAGWTFRL
PSGGDSFPQKYRLLAYCPLMCRAQGFPYAAVRFQPAGCSCLGPAEVDAVRSDTSSSPD
GASPDSTIQIIQLGSLQGSAFPKELLVLDRTSSGDQWSFSPRWITISIRLGAGLQGSLELE
GQTRSVSVLASGIPIKTPPVFIDASMAAIPKGFNIRGRLRISRDAGFFTSDLSQLSGQWQ
TVDAERNAECPQKCLEAGFSYAAMQTQTKRGWCKCALQLQNIDKYARRDSVKTGDIQ

VISLRRIGEKQLQVARLAELHRQAAIKAYKMIMQNPKWYRQVTVVSVPRTYGQNIGIA
SNPASCFKGCATSNFNYAFYTVYGCLCSNDKSDVKVVRPRYRSLKYFAAGRTSLFYIGR
VNRLKPSGTAAVIKGRKGYLILRSPNGRVQTGRFMAASLLQSQAWRRLPAVPPLFCLQ
MCALRGYTYAAVGNSKTQHVTAQELHQQILLFRVWRSDSDKQVFMLSGLPKAGKMT
SVTVMRGSFLQFTFSGTSAARPIVLYLRSAPPAGWTFRLPSGGDSFPQKYRLLAYCPLM
CRAQGFPYAAVRFQPAGCSCLGPAEVDAVRSDTSSSPDGASPDSTIQIIQLGSLQGAAIP
KELLVLDRTASGDQWSFX

>CL1824.Contig2_Ba_mix
XLINDGPLELTDTSVPRERGSLEVIQLRAMPPLAGELTAISYSVFEFIASYYRVIVNNDIV
PAGFSVSQPAGAWTSLGQMTISECIDTCSRESDLFLMVRTVDNVTSCWSAPLVDMSLL
RQQFPSVSGEYQVLDLSVMKIGTKITVTHSAGFNVSSTGIKNGYRGYAVKMPDESEPSE
LTVYYTKSAPSSLQLFFMTEHYPGNCVASAMGMMCDVAIWNPSESEKPCQCGQLING
GPLGLTDTSVPDVAGYMEVIQLRAMQPPADFEGLSYTMYDLFAGYFRLIVNYKALAVR
FSGSEPAGNWKNLGHLSVSECLDAC

>Unigene5401_Ba_mix
PDQYAALIDDQSSNLTDPTVSAALDKSRQAQSMSTSSISSIRDGLYEGQSKLLVEIRDAL
GGSPSGKNLDDVITTMNALHNDLMHSVGTLVTAMSDTNAALQTIISKMDGGSKQDPF
REIGLLATDRPLHSGDPDTGAMYYDCTFDDAAYENLNRAIGIIRSTRTPDPDDVVVIRF
SGVGLSIYMGDTAPPHSPDIWLTPVDRAATIGADSQMMIMLSTYTKQLGHDSLYYKFI
VWGGGFMSLLRAVATNPNVAARVSIQKLGHLIANXACVNDCFFENLCTLFSNCRLSGC
SACLRVIVPAMVKRGRRGGPSRGRGRVSTIVIPLDREDHATDGAGNAILPANNTVYAA
VTGEHILSAAPTVNGVRPSYARIRKITLDTTTGAALTTAGMGYYQAWRQYTVDPKTD
DIGGSIINAANKRPIPGSAGRFNYRNFPRFLGLSLMGVERQDVTINDKEPIYFAGVVYPD
ATPKPAVMLHIRAEFGGVAFVDSREPEVHSTTPHFDTNYGPDFLTRDFLASAMLALG
MTLGVLRDGVIMCERV

>CL166.Contig1_Ba_mix
XSHRFGGNSAYRSDEAHAYAAINPVVSNVRRAVGVSYSQGAPIVRSVSVEGPAPIAYSA
AAPITYSGLGYAGYHL

>CL4124.Contig1_Ba_mix
PLTALLAVLLLLALVYWYGTKDFGSLEKLGVPVIKPYPFIGSILKPWESFCVSDDLQNA
RKYGRLWGLYEGPWAQLFVADVDMAKHIMIKDFDHFQDRPVLFDFEYGRDQMDMM
PADRWKVLRSLLAPALTTSGRLKAMTPTMQQCLDDACRRMETKLRDSPVIDAQADL
FGPLTLDVMAQFSFGMKVPDVADKNSVFVKHASAFGVPTDATPNPLITYILTSFPSLG
NRVAGRQLIPAFRYFLEMIRRSIKQRRAEGVHRPDLVGVIMDAIDNKVPTAEYRQLRL
TEEILLLQGAEMLMAAYDTVGSALNKSAYFLATEPEAAEEVAQEAQELDELTYESVAQ
LPKLEAFIKEVLRMAPLLVRHFRVCTRDWEYQGLRVPAGTSVTIPMSVFHMDEQLFP
EARQFRPARWLEEDAHRSHQYSWMPFGLGPHACLGARLAMVECKFFLAGFLRRYRF
LACDQTKFEYLPGSGLFAT
